# Porous Functional Nanomaterials for Continuous Flow Catalysis

**DOI:** 10.1007/s40820-026-02149-0

**Published:** 2026-03-23

**Authors:** Yingguo Li, Hao Chen, Zhiyao Li, Minghao Liu, Mengmeng Fu, Xiaoyi Yue, Danfeng Jiang, Xiao Chen, Chao Yu, Wei Gong

**Affiliations:** 1 School of Environmental and Chemical Engineering, Jiangsu University of Science and Technology, Zhenjiang, 212003 Jiangsu People’s Republic of China; 2 School of Chemistry and Chemical Engineering, Frontiers Science Center for Transformative Molecules and State Key Laboratory of Metal Matrix Composites, Shanghai Jiao Tong University, Shanghai, 200240 People’s Republic of China

**Keywords:** Porous materials, Continuous flow, Process intensification, Photocatalysis, Heterogeneous catalysis

## Abstract

With the advancement of green chemistry and process intensification, continuous flow technology has emerged as a powerful tool in the manufacturing of fine chemicals and pharmaceuticals. Owing to their highly regular porous architectures, diverse chemical compositions, and excellent catalytic activity, porous materials have proven to be ideal supports and catalytic platforms for continuous flow catalysis. This review systematically summarizes the recent progress in the design and application of porous materials in continuous flow catalysis, with a focus on several major structural categories, including metal–organic frameworks, covalent organic frameworks/polymers, cages, porous silicates, monoliths, and polymeric carbon nitrides. It also covers various reactor types, including fixed bed, packed bed, and microreactors. Special emphasis is placed on elucidating the relationships among pore structure, electronic structure, active sites, and reaction–diffusion kinetics of porous catalysts within flow reactors. Their practical applications are outlined in areas such as selective catalysis of small molecules, photocatalysis, photothermal catalysis, and multistep cascade reactions in bioconversion processes. Furthermore, focusing on the technical challenges encountered during the industrial scale-up of continuous flow systems based on porous catalysts, this review examines key issues such as insufficient precise control over structure and function, limitations in the compatibility of particle and overall morphology design, difficulties in regulating low-pressure-drop fluid dynamics, and the challenge of maintaining high catalytic stability over extended operation. It also provides a systematic analysis of potential solutions to these problems. Finally, current challenges and future directions in the field are discussed, underscoring the pivotal role of porous materials in flow chemistry. It is hoped that this review will stimulate further research on the application of porous materials in continuous flow catalysis and facilitate the rational design of novel heterogeneous porous catalysts for industrial applications.

## Highlights

- This review provides a comprehensive summary of the latest advances in the application of porous materials in continuous flow catalysis.
- This review is categorized according to the properties of porous materials and also according to different continuous flow catalytic reactions.
- This review also examines the comparative advantages and disadvantages of batch reactors versus continuous flow reactors and discusses potential future developments in porous catalyst-based continuous flow systems.

## Graphical abstract

**Figure:**
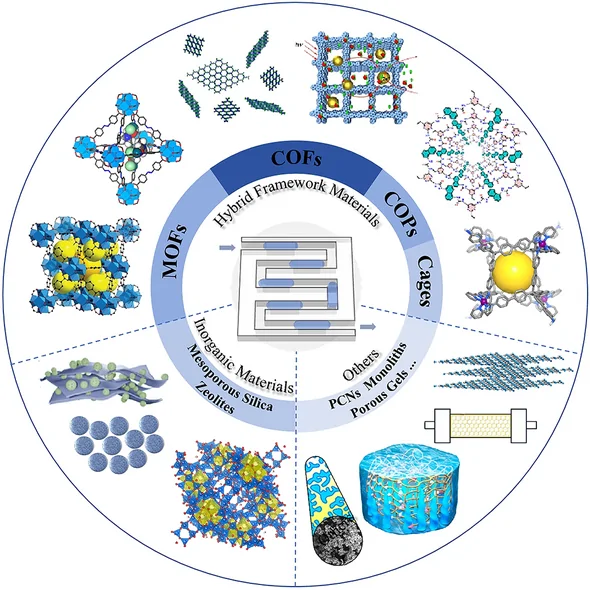

## Introduction

Continuous flow technology, as a paradigmatic manifestation of process intensification in modern chemical engineering, is fundamentally changing production methodologies in traditional chemical manufacturing [1]. In contrast to conventional batch reactors, continuous flow reactors achieve intrinsic enhancement of mass and heat transfer processes, precise regulation of reaction parameters, significant improvement in process safety, and seamless scalability from laboratory to industrial production through controlled directional transport and transformation of reactants across immobilized catalyst surfaces or within porous channels [2–5]. This technology provides an ideal platform for establishing green, efficient, and intelligent modern chemical production processes. It should be emphasized that the overall performance of continuous flow catalytic systems depends not only on reactor structural design but, more critically, is constrained by the microstructure and physicochemical properties of the catalyst [6].

Given this context, porous materials have demonstrated exceptional adaptability and broad application prospects owing to their unique structural advantages. Representative materials in this category include metal–organic frameworks (MOFs), covalent organic frameworks/polymers (COFs/COPs), cages and mesoporous silica, with the fundamental characteristics of exceptionally high specific surface areas, regularly tunable pore architectures, and directionally modifiable functional sites [7, 8]. These attributes establish a robust physicochemical foundation for maximizing the exposure of catalytic active centers, effectively reducing mass transfer resistance, and even enabling molecular size-based shape-selective catalysis [9, 10]. Upon integration of porous materials as catalysts or supports within continuous flow systems, their meticulously engineered hierarchical pore architectures constitute a distinctive “nanoscale transport network”. This sophisticated structure not only guarantees efficient and orderly contact between reactants and active sites, leading to enhanced reaction kinetics and selectivity but also imposes spatial confinement on reaction intermediates through nanoconfinement effects, thereby enabling precise manipulation of reaction pathways [11–13]. This feature proves particularly critical for complex system separations and tandem catalytic reactions. Moreover, through rational framework design or post-synthetic modification strategies applied to porous materials like MOFs and COFs, their catalytic performance, structural integrity, and operational adaptability in continuous flow environments can be further optimized [14–16].

It should be noted that while traditional inorganic porous materials (e.g., zeolites, mesoporous silica-alumina materials) have been widely employed in industrial continuous flow catalysis, this review focuses on emerging porous materials with highly designable pore architectures and tunable surface functionalities, such as MOFs, COFs/COPs, and cage-based structures. This emphasis aims to elucidate the intrinsic relationship between structural programmability of materials and process intensification in continuous flow systems, and to explore how rational material design enables precise control over catalytic reaction pathways, thereby highlighting recent advances and future potential in this interdisciplinary frontier.

This review systematically evaluates recent advancements in emerging porous materials for continuous flow catalysis. Firstly, the structural characteristics of various porous materials and their immobilization strategies within continuous flow systems were examined. Subsequently, the discussion focuses on pioneering applications across strategic domains including fine chemical synthesis, asymmetric catalysis, environmental remediation, and energy conversion. Finally, it will conduct an in-depth analysis of the critical challenges facing this technological framework, while offering a perspective on its future development directions. Through this systematic review of the interdisciplinary field, it is hoped to provide theoretical references and technical insights for advancing the greening and intelligent transformation of modern chemical processes.

## Overview of Continuous Flow Technology

Continuous flow technology, as an innovative alternative to traditional batch reactors, is increasingly demonstrating its exceptional value in modern synthetic chemistry and catalysis. The core of this technology lies in the continuous movement of reaction materials through fixed channels, where they interact with catalysts immobilized in a fixed bed, thereby achieving continuous transformation from reactants to products [17]. Compared to classical “tank-type” batch operations, the continuous flow mode, by virtue of its unique engineering principles, endows chemical reaction processes with a series of remarkable advantages [18, 19].

Firstly, continuous flow reactors enable highly efficient mass and heat transfer due to their exceptionally high surface-to-volume ratio. This not only effectively controls heat accumulation in strongly exothermic reactions, preventing side reactions and catalyst deactivation, but also significantly increases the reaction interface in catalytic reactions involving multiphase systems, thereby accelerating the reaction process [20]. Secondly, continuous flow systems provide unparalleled precision in process control. By precisely adjusting the flow rate of materials, the residence time of the reaction can be strictly controlled, which is crucial for suppressing the decomposition of unstable intermediates and improving the selectivity of complex reactions, thereby enabling precise regulation of reaction pathways [21]. Furthermore, continuous flow technology is inherently highly scalable. The transition from laboratory-scale process development to industrial-level production typically requires simple “numbering-up” rather than traditional “scale-up” significantly shortening the development-to-production timeline and ensuring process reproducibility and product consistency [22]. Finally, this technology is highly compatible with online analytical detection techniques, paving the way for process automation, intelligentization, and real-time monitoring [4, 23].

## Hybrid Frameworks Materials for Continuous Flow Catalysis

### MOFs for Continuous Flow Catalysis

MOFs are architectured through robust coordination bonds between metal-containing nodes (metal ions or clusters) and multitopic organic linkers. Guided by the principles of reticular chemistry, porous frameworks with tailored structures and functionalities can be rationally designed and synthesized. The building blocks of metal nodes and organic ligands allow precise functional modulation by varying their chemical nature and connectivity. Owing to their ultrahigh surface area, finely tunable pore environments, and customizable active sites, MOFs have emerged as a highly promising platform in heterogeneous catalysis.

Transitioning MOF-based catalysis from conventional batch reactors to continuous flow systems represents a critical step toward industrial application and process intensification. The continuous flow mode not only improves mass and heat transfer efficiency, leading to enhanced reaction rates and selectivity, but also addresses key limitations of batch processes, such as difficult recovery, nanoparticle leaching, and rapid deactivation of MOF catalysts, through the inherent immobilization of the catalyst. This integration substantially boosts the overall stability and spatiotemporal yield of the catalytic process.

#### Continuous Flow Catalytic Reaction of MOFs

**Fig. 1:**
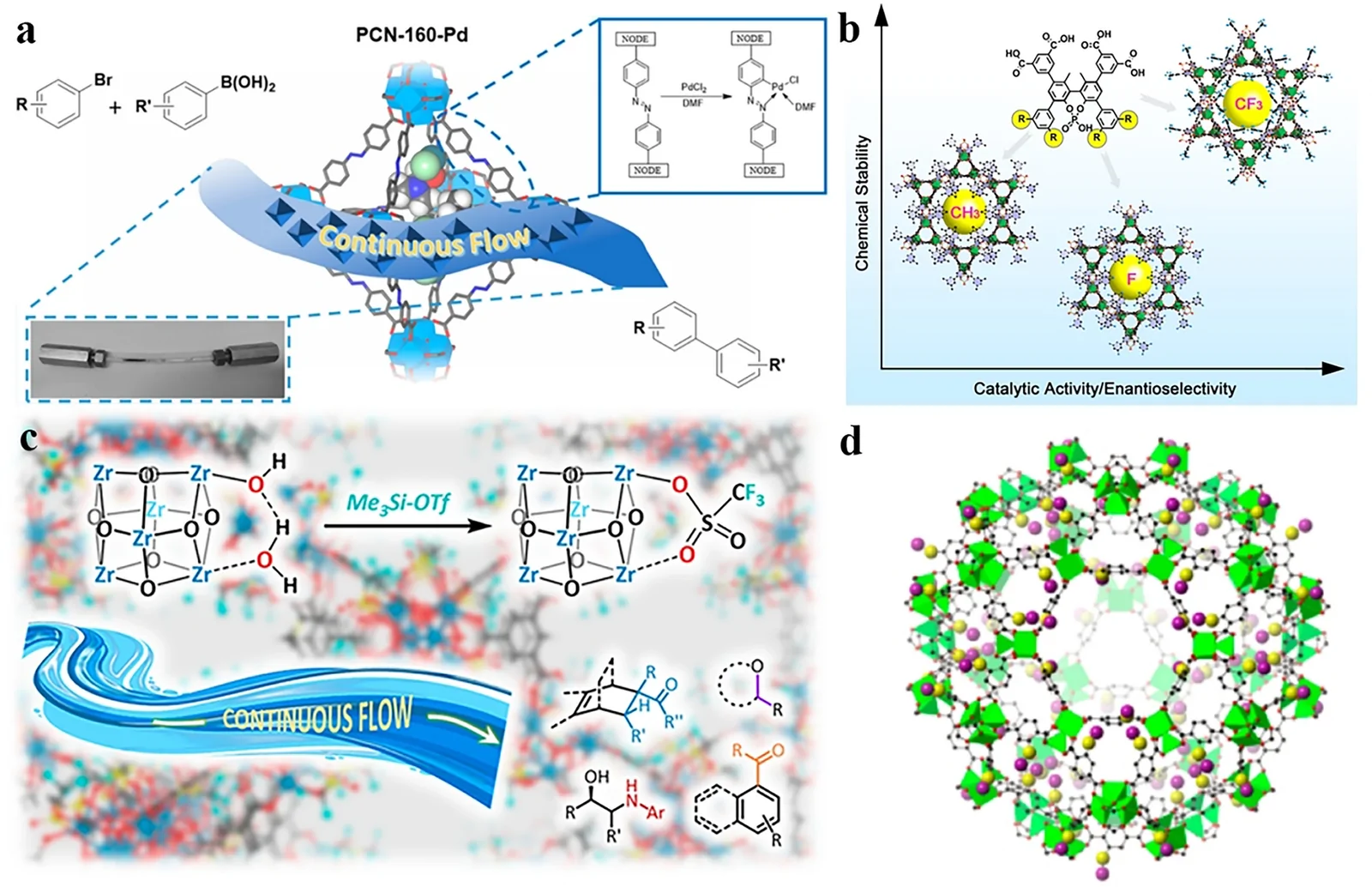
MOFs for continuous flow catalysis. **a** Site-isolated azobenzene-containing metal–organic framework for cyclopalladated catalyzed Suzuki–Miyuara coupling in flow [26]. Copyright 2021, American Chemistry Society. **b** Boosting chemical stability, catalytic activity, and enantioselectivity of metal–organic frameworks for batch and flow reactions [27]. Copyright 2017, American Chemistry Society. **c** Strongly Lewis acidic Zr-MOFs and ZrOTf-BTC for continuous flow catalysis [28]. Copyright 2019, American Chemistry Society. **d** The model of MIL-101-Cr-SO_3_H·Al(III) [30]. Copyright 2015, American Chemistry Society

Precise modulation of the pore size, crystal dimensions, surface functional groups, and the introduction of single-atom active sites in MOFs can enhance their catalytic performance and stability in continuous flow systems, providing effective strategies to address key challenges such as mass transfer limitations and catalyst deactivation. Madrahimov et al. [24] developed a catalyst based on the MOF NU-1000, modified with (bpy)Ni^II^ coordination sites, suitable for gas-phase ethylene dimerization. The material features a large pore size of 31 Å, which facilitates rapid diffusion of reactants and products, thereby overcoming the mass transfer limitations commonly associated with narrow-pore MOF catalysts. Under continuous flow conditions, the catalyst achieved a conversion rate exceeding 95%. However, catalyst deactivation was observed, primarily attributed to the coverage of active sites by polyethylene deposits. In terms of catalyst morphology and functional group regulation, they also studied the performance of UiO-66 in catalyzing the hydrolysis of ethyl paraoxon in a flow microreactor and found that crystal size and functional groups significantly impacted activity. Notably, the 14 nm small-sized UiO-66-NH_2_ exhibited the best activity within the particle range of 125–250 μm and remained stable during 18 h of continuous flow testing, without experiencing high pressure drops or bed channeling issues [25]. Moreover, they also constructed a site separated Pd single-atom catalyst PCN-160-Pd by post synthesis modification to coordinate palladium in PCN-160 containing azobenzene ligand (Fig. 1a). The catalyst continuously catalyzed Suzuki–Miyaura coupling reaction for 12 h in microfluidic reaction, with a stable yield of 85%. The coupling reaction yields of various aryl bromides and phenylboronic acid exceeded 92% without palladium leaching, demonstrating excellent catalytic activity and repeatability [26].

**Fig. 2:**
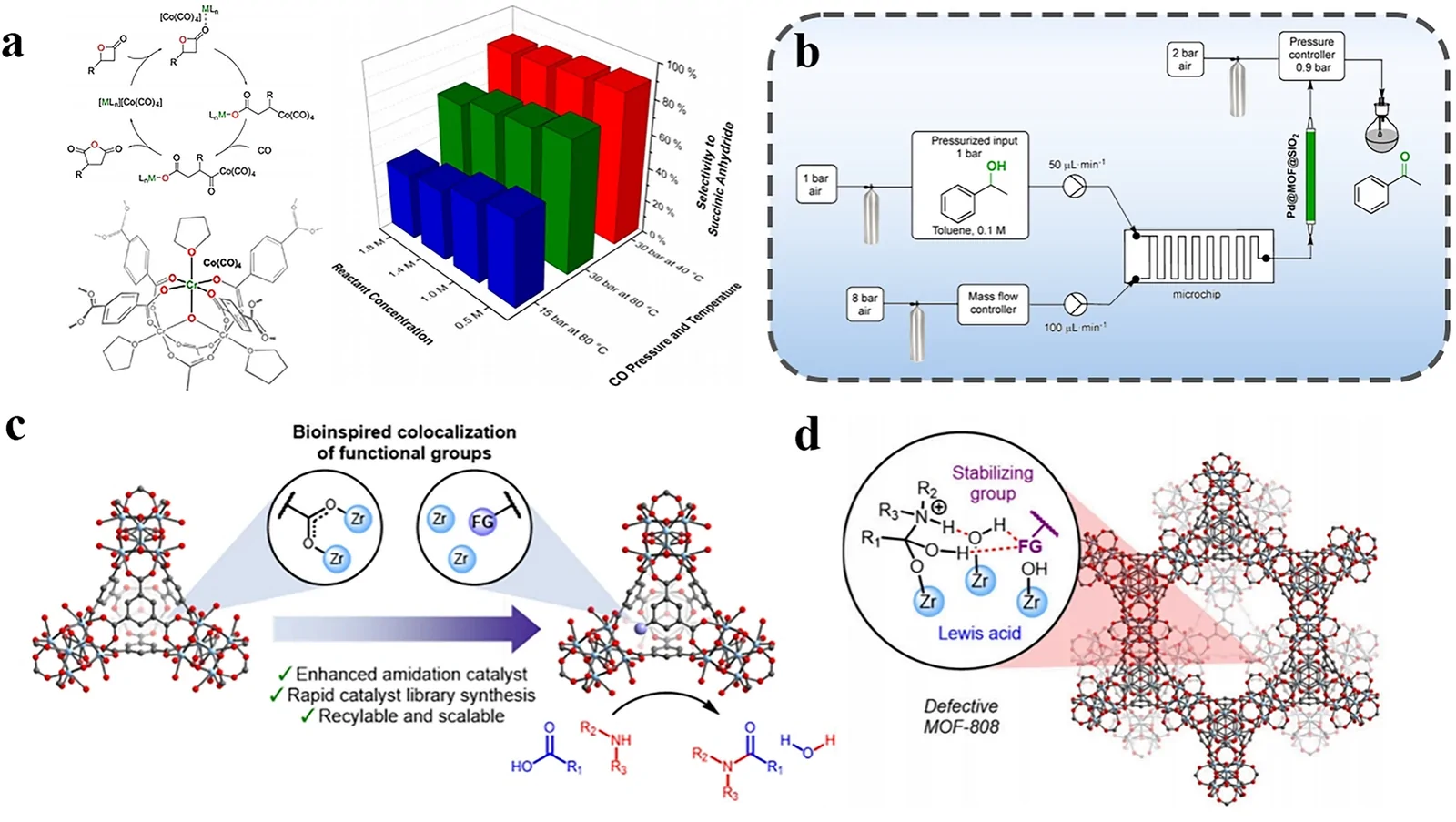
MOFs for continuous flow catalysis. **a** Selectivity to succinic anhydride as a function of the reaction conditions for batch carbonylation of *β-*butyrolactone by Co(CO)_4_ ⊂ Cr-MIL-101. Catalyst loaded at 0.5 Co mol% to the substrate in toluene and allowed to react for > 75% conversion of the substrate under all conditions tested [31]. Copyright 2018, American Chemistry Society. **b** Instrumental setup for aerobic oxidation under continuous flow [32]. Copyright 2014, American Chemistry Society. **c** Defect-engineered metal–organic frameworks as bioinspired heterogeneous catalysts for amide bond formation. **d** Co-localization of acid catalyst with synergistic functional groups within MOF [36]. Copyright 2024, American Chemistry Society

Cui et al. [27] demonstrated that chiral MOFs with the structural formula (Mn_2_L(H_2_O)_2_) can serve as effective catalysts for the enantioselective Friedel–Crafts reaction between pyrrole and nitroalkenes. In both batch and flow reaction systems, the CF_3_-functionalized MOF exhibited outstanding reactivity, selectivity, and recyclability, affording high yields and enantioselectivities in the alkylation of indoles and pyrrole with a range of ketoesters or nitroalkenes (Fig. 1b). Lin et al. [28] developed a highly Lewis acidic MOF, denoted as ZrOTf-BTC, through a two-step transformation of the secondary building units (SBUs) in MOF-808 (Fig. 1c). They further fabricated a ZrOTf-BTC@SiO_2_ composite for application in continuous flow catalysis. Under flow conditions, this composite exhibited exceptionally high turnover numbers (TONs) of 1600 for the Diels–Alder reaction, 2700 for epoxide ring opening, and 326 for Friedel–Crafts acylation. Das et al. [29] investigated the catalytic dehydration of 2-propanol over UiO-66 in a flow reactor and observed an initial increase in catalytic activity, attributed to the removal of adventitious formate and acetate ligands from the nodes and their replacement with hydroxyl groups generated in situ from water produced in the dehydration. Subsequently, catalytic activity declined and selectivity shifted as the node ligand environment continued to evolve. Li et al. [30] designed and synthesized MIL-101-Cr-SO_3_H·Al(III), a framework integrating both Brønsted acid sites and Al(III) Lewis acid centers (Fig. 1d). This heterogeneous solid acid catalyst exhibited excellent performance in the fixed-bed benzylation of aromatic hydrocarbons with benzyl alcohol, surpassing several benchmark zeolite-based solid acid catalysts. Similarly, D. Park et al. [31] reported that the MOF catalyst Co(CO)_4_ ⊂ Cr-MIL-101 enables continuous synthesis of succinic anhydride in a packed-bed reactor, exhibiting a carbonylation activity of 1300 mol_Anhydride_ mol_Co_^−1^ over 6 h on stream at room temperature using *β*-propiolactone as a substrate (Fig. 2a).

Pascanu et al. [32] employed MIL-88B-NH_2_ as a support to encapsulate palladium nanoparticles within its pores, followed by coating with SiO_2_ nanoparticles, forming a dual-protective Pd@MIL-88B-NH_2_@nano-SiO_2_ composite catalyst. This catalyst was evaluated for the aerobic oxidation of benzyl alcohol in a continuous flow microchannel reactor, demonstrating high activity and remarkable stability (Fig. 2b). It maintained structural integrity and catalytic performance for over 7 days at 110 °C without detectable palladium leaching, achieving a turnover frequency (TOF) of approximately 5 h^−1^. Mishra et al. [33] modified MIL-125-NH_2_ with a polydopamine (PDA) coating and subsequently immobilized AgPd bimetallic nanoalloys with an average particle size of 2.2 nm. The catechol and amino functional groups in the PDA layer enhanced the adsorption and anchoring of metal ions, effectively stabilizing the nanoparticles. The resulting AgPd@MIL-125-NH_2_-PDA catalyst was applied in various continuous flow reactions, including nitrobenzene reduction, aldehyde hydrogenation, formic acid dehydrogenation, and Suzuki–Miyaura coupling, exhibiting high catalytic activity and recyclability. After multiple reaction cycles, the catalyst retained its original morphology and metal loading, with the formic acid dehydrogenation TOF being twice that of unmodified MOF-supported monometallic catalysts.

Griffiths et al. [34] immobilized MIL-100(Sc) on a polymer-based spherical activated carbon (PBSAC) carrier to create a composite catalyst with good mechanical strength and geometric morphology, addressing the application limitations of MOF powder in flow reactors. After continuous operation for 9 h in the intramolecular cyclization reaction of ( ±)-vanillin, there was no decrease in activity, with an average conversion rate of 72.9%. The yield of ( ±)-isocalamanol reached 64.4%, and no catalyst breakage or reactor blockage occurred, demonstrating the potential of MOFs fixed on carriers for industrial continuous flow Lewis acid catalysis. Schlichte et al. [35] employed (Cu_3_(BTC)_2_) for the *N-*methylation of aromatic amines, the ring opening of epoxides, and the aldolization of aldehydes. By adjusting the flow rate under continuous flow conditions, they achieved a short residence time, which enhanced both catalytic efficiency and yield. The material’s high specific surface area and microporous structure contributed to improved reaction kinetics. Additionally, it demonstrated good mechanical and structural stability throughout the reaction, with no changes in particle size, thereby confirming its feasibility and optimization potential as a porous polymer material in continuous flow applications. These case studies demonstrate that MOFs can effectively adapt to continuous flow systems, achieving high activity, high selectivity, and long-term stable operation through various strategies such as chiral design, acid site engineering, composite material construction, carrier immobilization, and optimization of process parameters.

**Fig. 3:**
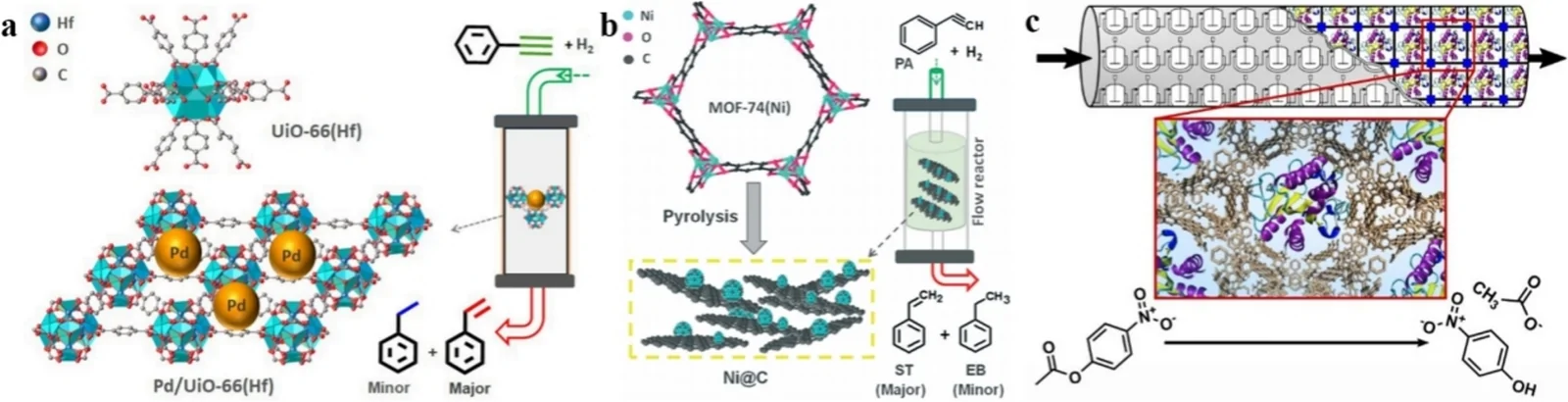
MOFs for continuous flow catalysis. **a** Continuous flow liquid-phase semi-hydrogenation of phenylacetylene over Pd nanoparticles supported on UiO-66(Hf) metal–organic framework [37]. Copyright 2023, Wily-VCH. **b** MOF-74(Ni) usage for semi-hydrogenation of phenylacetylene in a fixed-bed flow reactor [38]. Copyright 2022, Royal Society of Chemistry. **c** Schematic illustration of the biocatalytic process in the continuous flow reactor [40]. Copyright 2022, Wily-VCH

Utilizing defect engineering and metal-MOF hybridization strategies enables precise modulation of the microenvironment of active sites and metal–support interactions in MOFs, thereby achieving high activity, high selectivity, and excellent stability in continuous flow reactions. Ahmad et al. [36] co-localized Lewis acidic zirconium sites and pyridine *N-*oxide functionalized defect sites in MOF-808 through defect engineering and prepared MOF-808-py-Nox catalyst for continuous flow amide bond formation reaction (Fig. 2c, d). Density functional theory (DFT) simulation shows that pyridine *N-*oxide stabilizes zirconium sites through hydrogen-bonding network, synergistically enhances carboxylic acid activation, and the structure, specific surface area, and catalytic performance of the catalyst remain basically unchanged after five cycles. Swamy et al. [37] constructed a Pd/UiO-66 (Hf) catalyst by anchoring Pd nanoparticles on the outer surface of UiO-66, which was used for the semi-hydrogenation of phenylacetylene (PA) to styrene (ST) in a continuous flowing liquid phase. Under optimized conditions, a PA conversion rate of 99.0% and ST selectivity of 90.0% were achieved, with only slight deactivation after 4 h of continuous operation. Its superiority lies in the strong metal carrier interaction between Pd nanoparticles and MOF surface functional groups, effectively suppressing particle aggregation (Fig. 3a). The catalyst can be reused three times after hydrogen regeneration and its activity and structure remain stable. Bakuru et al. [38] prepared carbon-doped nickel-based catalysts using MOF-74(Ni) as a precursor through carbonization and hydrogen reduction of Ni@C. In a continuous flow fixed-bed reactor, nearly complete conversion (> 99%) of phenylacetylene was achieved, while the selectivity for styrene reached 92% ± 1%. The catalyst demonstrated stable operation for 13 h and maintained its activity after four regenerations (Fig. 3b). Characterization results indicate that carbon atoms embedded in the nickel lattice form a NiC_x_ structure, which reduces the crystallinity of nickel nanoparticles and enhances surface electron polarization. DFT calculations further suggest that carbon doping weakens the adsorption energy of styrene and increases the energy barrier for its further hydrogenation, thereby dynamically promoting high selectivity for semi-hydrogenated products.

The co-catalytic effect of MOFs on metal nanoparticle catalysis has garnered significant attention in recent years. Yoshimaru et al. [39] systematically investigated the performance of Pt nanoparticles loaded with various MOFs in catalyzing the hydrogenation of acetic acid to ethanol in a continuous flow system. Their findings indicated that MOFs containing amino groups exhibited a stronger affinity for acetic acid adsorption. Notably, Pt/MIL-125-NH_2_ achieved an ethanol yield of 31% at 200 °C in a fixed-bed reactor, surpassing the yield of traditional Pt/TiO_2_ by more than eight-fold. In situ infrared spectroscopy and DFT calculations demonstrated that MIL-125-NH_2_ effectively adsorbs acetic acid while weakly adsorbing ethanol, thereby significantly inhibiting the formation of the by-product ethyl acetate. Temperature-programmed desorption mass spectrometry (TPD-MS) and gas-phase tolerance tests further confirmed that the pore structure and functional groups of the MOF dictate the strength of substrate adsorption. This study employed arc plasma deposition to fabricate Pt/MOF composite catalysts, effectively mitigating the influence of size and loading factors, and confirming that the substrate adsorption support effect of MOFs is the primary contributor to catalytic activity.

In the field of biocatalysis, MOFs also demonstrate the potential for enzyme immobilization. Their tunable pore structures not only provide efficient mass transfer channels but also offer effective confinement protection for enzyme, thereby significantly enhancing the efficiency and stability of biocatalytic processes in continuous flow systems. Greifenstein et al. [40] loaded the esterase AaEST2 from thermophilic bacteria into the macroporous NU-1000 by diffusion method and constructed an enzyme @MOF composite material without chemical modification (Fig. 3c). For the first time, it was integrated into a high-performance liquid chromatography system to achieve continuous flow reaction; when catalyzing the hydrolysis of 4-nitrophenylacetate in aqueous phase, the space–time yield is as high as 1432 g L^−1^ h^−1^, which is one order of magnitude higher than traditional immobilized enzyme reactors. At the same time, the confinement protection effect of MOF increases the stability of immobilized enzyme by about 30 times; this system can also continuously synthesize isoamyl acetate in organic acetonitrile solvent, demonstrating excellent solvent tolerance. Molecular dynamics simulations show that the enzyme undergoes slight conformational changes after embedding into MOF pores, which partially reduces catalytic efficiency but significantly improves stability and operational flexibility, resulting in significant overall performance advantages.

#### Key Evaluation of the Advantages and Disadvantages of MOFs Materials in Continuous Flow Catalytic Reactions

*Advantages*: (I) High specific surface area and abundant active sites: MOFs exhibit specific surface areas reaching several thousand square meters per gram, thereby offering substantial spatial capacity for catalytic processes. The high density of active sites per unit volume of catalyst enhances the spatiotemporal yield of the reactor, facilitating reactor miniaturization and efficient production. (II) Tailorable framework and controlled porosity: Through the judicious selection of metal nodes and organic ligands, MOFs with precisely defined pore dimensions, geometry, and chemical microenvironments can be rationally designed and synthesized. Such well-defined porous architectures enable molecular sieving of reactants and products, thereby enhancing reaction selectivity. This property ensures the sustained acquisition of high-purity products in continuous flow systems. Furthermore, the unique confined environment within the pores can concentrate reactants, stabilize reactive intermediates, accelerate reaction rates, and modulate reaction pathways. (III) Well-defined and uniform active sites: The active sites in MOFs may consist of coordinatively unsaturated metal centers, functionalized organic ligands, or species introduced via post-synthetic modification, such as metal complexes or nanoparticles. These sites are typically uniformly distributed throughout the crystalline framework. The homogeneity of active sites contributes to consistent and reproducible catalytic performance, which is essential for the long-term operational stability of continuous flow processes and stringent product quality control. (IV) Multifunctional cooperative catalysis: Diverse catalytic sites, including acidic, basic, redox-active, and chiral centers can be simultaneously incorporated into a single MOF framework. This multifunctionality enables the execution of multistep tandem reactions in a continuous flow reactor via a one-pot strategy, eliminating the need for intermediate separation. Such integration streamlines the process flow, improves atom economy, and enhances overall production efficiency.

*Disadvantages*: (I) Limited chemical and mechanical stability: Numerous MOFs exhibit structural vulnerability under aqueous, acidic, alkaline, or elevated temperature conditions, where coordination bonds may undergo hydrolysis or degradation. Their crystalline frameworks are often fragile and possess restricted mechanical strength, thereby constraining their practical applicability (Table 1). (II) Instability of active sites: Coordinatively unsaturated metal sites are prone to adsorption of impurities, leading to deactivation. Meanwhile, embedded nanoparticles may migrate, aggregate, or leach under thermal stimuli during catalytic reactions. In prolonged continuous operation, the loss or inactivation of active sites results in progressive deterioration of catalytic performance, which shortens the service life of the catalyst and elevates operational costs. (III) Mass transfer limitations: Although MOFs feature open porous structures, their pore systems remain predominantly microporous (pore width < 2 nm). In reactions involving bulky molecular reactants or requiring rapid kinetics, intracrystalline diffusion often becomes the rate-determining step. Despite high apparent flow rates, the slow molecular transport within micropores substantially restricts the overall process efficiency. (IV) Challenges in Cost and Scalability: The synthesis of many high-performance MOFs relies on costly organic ligands (such as porphyrins or chiral linkers) and metal precursors (such as Zr or Hf salts). These material expenses, coupled with complexities in large-scale synthesis, pose significant barriers to their industrial production and practical implementation.

**Table 1:** Lifetime under different pH levels and temperatures of MOF materials

MOF materials	pH stability	Temperature stability	Refs
UiO-66-NH_2_	Retains crystallinity after immersion in 1 M HCl or NaOH aqueous solution for 3 days, with a decrease in BET surface area of less than 10%	Thermally stable up to 380 °C; no structural change observed after catalytic cycles at reaction temperatures between 20 and 90 °C	[25]
Chiral MOFs with [Mn_2_L(H_2_O)_2_]	Loses crystallinity after 6 h in water; undergoes amorphous transformation after less than 3 h in solutions of pH = 4 or 8	Stable up to 380 °C; exhibits poor stability under catalytic conditions at 0–30 °C	[27]
Cu_3_(BTC)_2_	Decomposes within days at room temperature in CH_2_Cl_2_; catalytic Lewis acid sites are completely passivated in THF, indicating poor solvent tolerance and indirect sensitivity to acid/base conditions	Framework decomposition occurs in solution above 40 °C; reduction of Cu^2+^ by aldehyde substrates leads to visible color change	[35]
MIL-125-NH_2_	Decomposes in acetic acid, indicating instability in acidic medium	Structural degradation begins above 250 °C	[39]

In summary, MOFs have shown significant advantages in continuous flow catalysis, mainly reflected in their highly ordered pore structure and controllable chemical environment, which can provide high density and uniformly distributed active sites, thereby significantly enhancing mass transfer efficiency and reaction rate. At the same time, their modular composition characteristics facilitate the precise design of catalyst functions. However, it still faces key challenges in practical continuous flow applications, mainly including the insufficient stability of most MOFs materials under hydrothermal or mechanical stress conditions, which can easily lead to structural collapse and loss of activity, and their inherent microporous characteristics may limit the diffusion of substrate molecules, especially when dealing with large molecule reactions. At present, there are still certain difficulties in the large-scale synthesis of MOFs catalysts with good mechanical strength and formability, which restricts their feasibility for industrial scaling up.

### COFs/COPs for Continuous Flow Catalysis

#### Continuous Flow Catalytic Reaction of COFs

To specifically address the key limitations of MOFs in continuous flow catalysis, such as structural stability, mass transfer constraints, and scalability, research focus is shifting toward COFs, which offer superior intrinsic stability and enhanced structural designability. COFs, by virtue of their distinctive structural advantages, are infusing new vitality into heterogeneous catalysis. Their integration with continuous flow technology, a paradigm championed by modern chemical engineering, demonstrates considerable potential to revolutionize conventional catalytic processes. Composed of organic building blocks interconnected via robust covalent bonds, COFs are crystalline porous polymers characterized by highly ordered pore channels, substantial specific surface areas, and a pore microenvironment that can be precisely tailored. The synergy between COFs and continuous flow technology is mutually reinforcing. Continuous flow operation amplifies the intrinsic material advantages of COFs, while the incorporation of COFs as catalysts simultaneously drives the evolution of continuous flow systems toward enhanced efficiency, stability, and sustainability. The exceptional structural stability and insolubility of COFs underpin the mechanical integrity and catalytic activity retention of the packed catalysts during prolonged continuous operation. Furthermore, the designable pore architecture of COFs enables precise modulation of their hydrophilicity, hydrophobicity, or chiral microenvironments. This allows for the creation of optimized nanoreactors for specific catalytic transformations, such as asymmetric synthesis, photocatalysis, or cascade reactions, that are challenging to realize using traditional porous materials. The profound integration of COFs with continuous flow catalysis not only offers a material-based strategy to address long-standing limitations in mass transfer and catalyst stability within traditional heterogeneous catalysis, but also paves a novel pathway toward efficient, energy-conserving, automated, and readily scalable green synthesis processes. This convergence represents a significant direction for the intensification of future chemical manufacturing.

##### COFs for Continuous Hydrogen Peroxide Synthesis

**Fig. 4:**
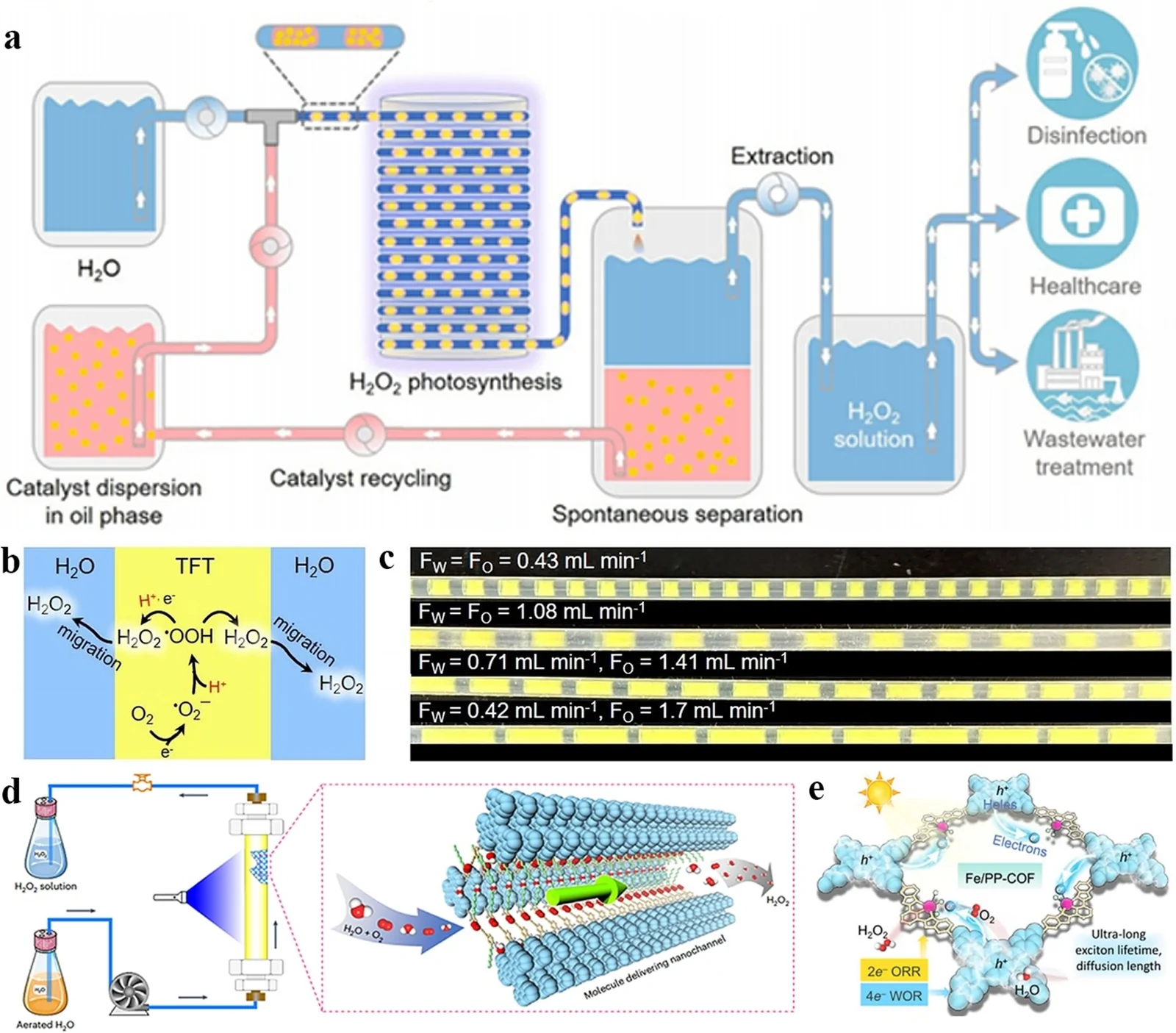
COFs for continuous flow catalysis. **a** Biphasic fluid system that enables continuous H_2_O_2_ photosynthesis, separation, and extraction. **b** Schematic illustration of H_2_O_2_ formation in TFT and migration across the oil–water interfaces. **c** Typical images of oil–water segments formed inside tubular flow channels at different flow rates and F_W_/F_O_ ratios [41]. Copyright 2024, Springer Nature. **d** Schematic of manufacturing H_2_O_2_ solution via continuous photocatalysis in a flow reactor. Inset: image for converting water and O_2_ into H_2_O_2_ through the 1D nanochannel [49]. Copyright 2024, Springer Nature. **e** Proposed mechanism for H_2_O_2_ production on Fe/PP-COF [50]. Copyright 2025, Wily-VCH

In traditional photocatalytic hydrogen peroxide (H_2_O_2_) synthesis research, most systems use single-phase batch reactors, which possess problems such as low yield, discontinuous operation, and difficult product separation, limiting their practical applications. In recent years, COFs materials have shown significant advantages in continuous flow photocatalytic synthesis of H_2_O_2_ due to their controllable electronic structure and pore environment. In redox reactions, COFs achieve efficient catalysis by regulating electron transfer and the stability of active intermediates (Table 2). Shao et al. [41] developed a perfluoroalkyl modified superhydrophobic PF-BTTA-COF, synthesized by [4 + 3] Schiff base reaction, and used unreacted aldehyde groups to graft perfluoroalkyl chains to stably disperse them in *α, α, α*-trifluorotoluene oil phase (Fig. 4a). In the constructed two-phase flow reaction system, a stable segmented flow is formed between the aqueous phase and the catalytic oil phase, and H_2_O_2_ rapidly migrates across the interface to the aqueous phase after being generated in the oil phase, achieving in situ separation and continuous collection of products. By optimizing parameters such as flow rate, oil–water ratio, and adding benzyl alcohol as a hole sacrificial agent (Fig. 4b), the system achieved a yield of 968 μmol h^−1^, with product concentration adjustable within the range of 2.2 to 38.1 mM. The resulting H_2_O_2_ solution can be directly used for bacterial inhibition and organic dye degradation. These two examples verified abilities of the F-modified COFs for continuous H_2_O_2_ production.

**Table 2:** Comparison and analysis of hydrogen peroxide yield with different catalysts

Catalyst system	Reactor type	H_2_O_2_ yield (μmol g^−1^ h^−1^)	Key advantage	System characteristics	Refs
PF-BTTA-COF	Oil–water biphasic system	968	Superhydrophobic catalyst, spontaneous H_2_O_2_ extraction	Microreactor, TFT as oil phase, continuous flow, no sacrificial agent	[41]
TAPT-FTPB-COF	Flow-type photocatalytic microreactor	3780	Enhanced O_2_ adsorption, high SCC efficiency	Quartz microreactor, continuous flow, efficient sterilization & coupled reaction	[42]
COF-TPDB-NO_2_	Flow system	1400	Strong built-in electric field, high charge separation	Custom flow setup, 70 h operation, 4.4 L of solution collected	[43]
TFBP-DHBD-COF	Biphasic Microreactor (on Al_2_O_3_ balls)	1456	Proton relay, enhanced ORR & WOR	Annular pipe reactor, natural sunlight, continuous sterilization	[47]
ACOF-S-EtOH	Continuous flow reactor	5440	Enzymatic click PSM strategy, high stability & activity	Fixed-bed reactor, metal-free, environmentally friendly	[48]
Fe/PP-COF	Biphasic flow reactor	1556	Ultralong exciton lifetime, high stability	Custom biphasic system, BA as organic phase, continuous production	[50]
COP-2	Coiled tube Photomicroreactor	5446	Efficient gas–liquid–solid mass transfer, no sacrificial agent	Coiled tube reactor, visible light, continuous 72 h run	[65]
PP-COP-4	Panel reactor	2758	Synergistic dual active sites enhanced by electron-donating groups	*λ* = 365 nm, in pure water and without metal cocatalysts	[66]

Building on the advancements in interface engineering, research has progressed toward precise modulation of the intrinsic electronic structure of materials. The strategy of atomic engineering has been implemented in COFs to enhance their photocatalytic H_2_O_2_ production performance. Liu et al. [42] constructed TAPT-FTPB-COF through molecular engineering to modulate the electronic structure. By incorporating pyrrole-type sulfur atoms, they induced localized electronic symmetry breaking, which improved oxygen adsorption and enhanced the separation efficiency of photogenerated charge carriers. Under continuous flow microreactor conditions, a photocatalytic conversion efficiency of 1.22% was achieved, and the system was successfully applied for bacterial inactivation and coupling reactions. Su et al. [43] introduced nitro functional groups into COF-TPDB (denoted as COF-TPDB-NO_2_), generating a strong built-in electric field that facilitated electron–hole separation and strengthened O_2_ adsorption. In a continuous flow reactor, a steady H_2_O_2_ concentration of 0.59 mM was maintained for 70 h without significant loss of catalytic activity. Wang et al. [44] developed an ionic COF material, JUC-660, by incorporating quaternary ammonium groups into the framework. This modification redirected the oxygen reduction reaction pathway from a four-electron to a two-electron process, leading to an H_2_O_2_ selectivity of 86.6%. In a flow-cell setup, the material exhibited sustained operation for over 85 h with a production rate exceeding 1200 mmol g^−1^ h^−1^. The material also demonstrated stable performance under acidic conditions and was effectively employed in electro-Fenton processes for organic dye degradation. Hou et al. [45] synthesized two structural isomers, COF-BD1 and COF-BD2, and demonstrated that COF-BD2 with a convergent charge transfer pathway achieved a remarkable H_2_O_2_ production rate of 5211 μmol g^−1^ h^−1^ in the absence of sacrificial agents. This catalyst retained high photocatalytic activity across a broad pH range and in diverse real water matrices, and was successfully immobilized in an outdoor continuous flow apparatus (50 cm × 30 cm × 5 cm). Moreover, it enabled efficient H_2_O_2_ generation under natural sunlight for in situ water disinfection and pollutant degradation. Ma et al. [46] developed a two-dimensional COF-S-OH based on acylhydrazone bonding, introducing benzothiophene units and hydroxyl functional groups to enhance light absorption, electron donor–acceptor effect, and proton conductivity. Employing this material to a plate serpentine continuous flow reactor, it can efficiently degrade organic pollutants under natural light. Under non aeration conditions, 1.9 L of wastewater can be treated within 3 days, and the degradation efficiency is positively correlated with light intensity.

While optimizing the electronic structure of materials, research also focuses on enhancing key reaction kinetics through the design of functional groups. Sun et al. [47] proposed the incorporation of a proton reservoir into a COF to enhance the photosynthetic production of H_2_O_2_. They designed and synthesized two COF variants of TFBP-DHBD-COF and TFBP-BD-COF. The hydroxylated COF exhibited an H_2_O_2_ production rate of 1444.0 μmol g^−1^ h^−1^, which is 3.3 times higher than that of the hydroxyl-free material. The researchers also developed a streamlined continuous flow reactor by immobilizing the COF on Al_2_O_3_ microspheres. This system achieved sustained H_2_O_2_ generation at a rate of 50.0 μmol h^−1^ under natural sunlight in lake water, accompanied by efficient sterilization with a 100% inactivation rate (Fig. 4c). Zuo et al. [48] employed a horseradish peroxidase-catalyzed click reaction in aqueous medium at room temperature to graft 2-hydroxyethylthio and ethylthio groups into allyloxy-functionalized COF pores, achieving mild and eco-friendly functionalization. This modification optimized the adsorption of oxygen reduction intermediates and improved proton supply, leading to an H_2_O_2_ production rate of 5440 μmol g^−1^ h^−1^ in pure water without sacrificial agents, with an apparent quantum efficiency of 13.3%. A continuous flow photocatalytic reactor based on ACOF-S-EtOH steadily produced H_2_O_2_ for 58 h, yielding a cumulative output of 2.5 L. With a deepened understanding of the synergistic interplay between active sites and mass transfer processes, research is advancing toward the development of hierarchical structural design and system integration. Chen et al. [49] designed a donor–acceptor COF system using hexavalent triphenylbenzene as the electron donor and diphenylbutadiyne as the electron acceptor. By incorporating hydrophilic side chains into the pore walls, the researchers tuned the hydrophilicity–hydrophobicity balance to promote capillary-driven transport of water and dissolved oxygen (Fig. 4d). The optimized material, TP-DPBD30-COF, achieved an H_2_O_2_ production rate of 7.2 mmol g^−1^ h^−1^ in batch reactions, with an apparent quantum efficiency of 18.0%. In a continuous flow configuration, using air-saturated water and visible light as inputs, the system enabled stable and continuous generation of H_2_O_2_ solution, accumulating over 15 L during 336 h of operation. Fang et al. [50] anchored iron single atoms within a PP-COF matrix via tridentate chelation, which enhanced the π-conjugation and extended the exciton lifetime to 296.75 ps. Using a biphasic flow reactor (Fig. 4e), they accomplished continuous photocatalytic H_2_O_2_ synthesis with a stable production rate of 1556 μmol h^−1^ over 60 h, without detectable leaching of single atoms.

##### COFs for the Continuous Flow Synthesis of Other Substances

**Fig. 5:**
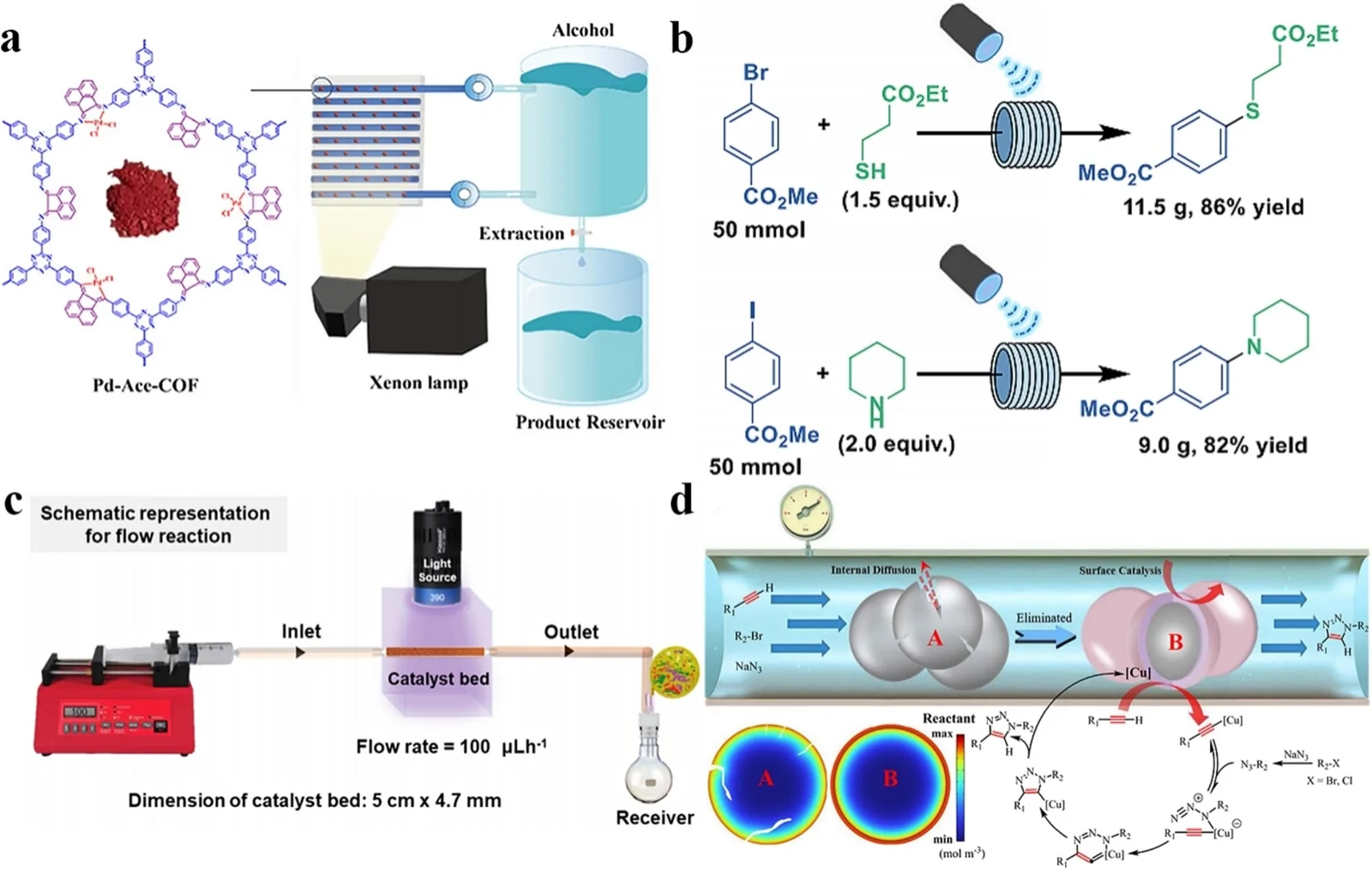
COFs for continuous flow catalysis. **a** Schematic of the visible-light-driven continuous flow system with Pd-Ace-COF [51]. Copyright 2025, Royal Society of Chemistry. **b** High-speed circulation flow enabled decagram-scale synthesis [52]. Copyright 2025, Springer Nature. **c** Late-stage functionalization of ketoprofen drug in batch and continuous flow reaction and schematic representation of continuous flow reaction [54]. Copyright 2024, American Chemistry Society. **d** Diagram of the CuAAC reaction mechanism in continuous flow and concentration distribution at the surface of solid phase (A) and porous particle phase (B) [56]. Copyright 2024, American Chemistry Society

COFs have been widely employed as heterogeneous catalysts for the continuous synthesis of organic compounds. For instance, Ma et al. [51] harnessed the synergistic interaction between the light-harvesting capability of Pd-Ace-COF and its atomically dispersed palladium sites to achieve visible-light-mediated C–C coupling in a continuous flow reactor, yielding biphenyl with up to 99% efficiency (Fig. 5a). In a recent contribution, Jiang et al. [52] developed a series of nickel-incorporated pyridyl-quinoline-linked COFs (Ni-PQCOFs) that function as efficient and stable metallaphotocatalysts. They further implemented a custom high-speed circulation flow system to enable decagram-scale synthesis of organic compounds via Ni@PQ-COF-OMe-based heterogeneous photocatalysis (Fig. 5b). Jati et al. [53] developed a nickel-embedded π-conjugated covalent organic framework (Ni@COF1) for visible-light-driven aromatic Finkelstein and retro-Finkelstein reactions. Under continuous flow conditions on a gram scale, Ni@COF1 exhibited enhanced stability, achieving a chlorination yield of 81% for isoproterenol drug derivatives, compared to 77% in batch mode. This improvement can be attributed to the effective mitigation of catalyst deactivation in the continuous flow system, which optimizes contact time and flow kinetics, thereby eliminating issues such as nickel black formation commonly encountered in homogeneous catalysis. Furthermore, the same group reported the application of an anthraquinone-based COF (TpAQ) in photocatalytic decarboxylation and fluorination reactions [54]. In a gram-scale continuous flow setup, TpAQ achieved a fluorination yield of 82% for ketoprofen, surpassing the 77% obtained in batch operation. Moreover, the catalyst retained 80% of its initial activity after eight reaction cycles. Post-reaction characterization via PXRD and SEM confirmed that the crystalline structure and morphology of TpAQ remained intact, underscoring its high chemical stability. This methodology is applicable to other decarboxylation and halogenation reactions, generally leading to a 5%-10% yield enhancement under flow conditions compared to batch processes (Fig. 5c). These findings underscore the potential of continuous flow systems to enhance both reaction efficiency and catalyst durability, particularly in the context of large-scale synthesis. In another study, Ma et al. [55] reported a straightforward strategy for the direct synthesis of (S)-2-(2-chlorophenyl)-2-(6,7-dihydrothieno[3,2-c]pyridin-5(4H)-yl)acetonitrile ((S)-CIK) via a heterogeneous Strecker reaction catalyzed by a homochiral covalent organic framework. They also engineered a continuous flow-through system for its gram-scale production. Wang et al. [56] fabricated a silica gel-supported COF (SiO_2_@CuI-TpBpy) through the stepwise growth of TpBpy on SiO_2_, followed by chelation with copper(I), resulting in a stable, high-loading heterogeneous catalyst (Fig. 5d). This material was employed in a packed-bed reactor for three-component catalytic reactions under continuous flow, enabling the synthesis of rufinamide over 24 h with an 89% yield. Wang et al. [57] designed nitrogen-rich spherical COFs to anchor silver nanoparticles within their channels. The resulting microspheres exhibited high catalytic activity in the reduction of 4-nitrophenol (4-NP), achieving a permeation flux of 2000 L m^−2^ h^−1^ (LMH) and a reduction efficiency exceeding 99% in continuous flow operation.

**Fig. 6:**
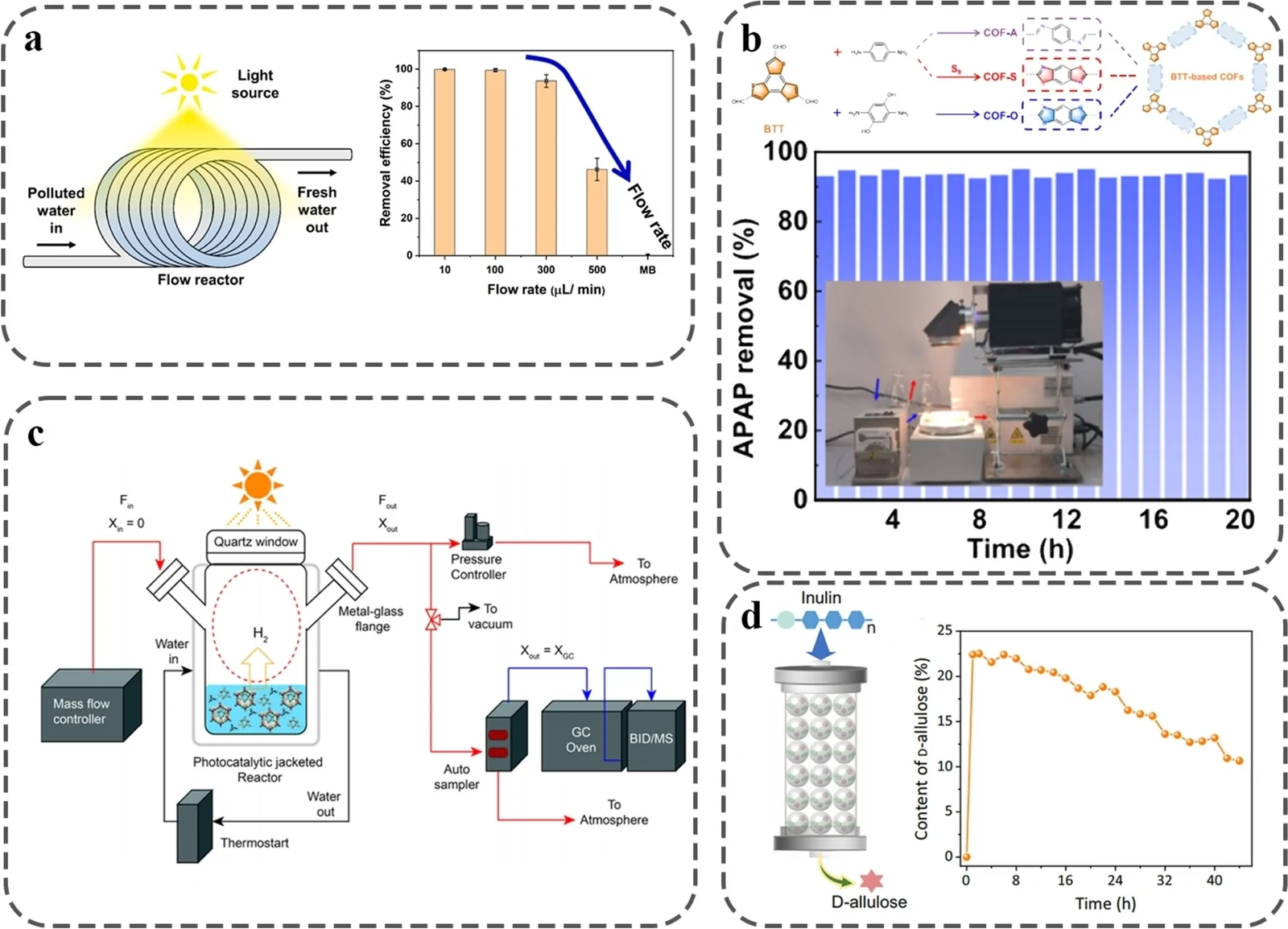
COFs for continuous flow catalysis. **a** Schematic representation of continuous flow system, and continuous degradation organic pollutants in glass flow reactor [59]. Copyright 2024, Elsevier. **b** Degradation performance of paracetamol by immobilized COF-S in a continuous flow reactor under visible-light irradiation for 20 h and in an enlarged reactor of 2 L under natural sunlight irradiation in 5 days [60]. Copyright 2024, Springer Nature. **c** Continuous flow photocatalytic reactor design. Schematic diagram of the designed continuous flow photocatalytic reactor system [61]. Copyright 2019, American Chemistry Society. **d** Schematic diagram of the continuous flow reaction for the production of D-allulose from inulin by INU-NH_2_&E-NH_2_@NKCOF-141 [62]. Copyright 2024, Springer Nature

Beyond the field of organic synthesis, COFs have also achieved significant results in continuous flow applications for environmental remediation, demonstrating the expanding potential of material multifunctionality. Jin et al. [58] utilized a partially fluorinated triazine-based COF (TP/TAPT-F) to achieve efficient hydrogen peroxide (H_2_O_2_) synthesis via synergistic two-electron oxygen reduction and four-electron water oxidation reactions. The fluorinated COF was subsequently immobilized on a polyvinylidene difluoride (PVDF) membrane through vacuum filtration and integrated into a continuous flow reactor for aqueous-phase applications. In a separate study, Gogoi et al. [59] employed mechanical pulverization to prepare COF powders with enhanced visible-light photocatalytic activity for environmental remediation. The improved performance of the pulverized COFs was attributed to their high dispersibility, efficient visible-light absorption, and superior charge carrier mobility (Fig. 6a). These materials also exhibited exceptional photocatalytic reduction of toxic hexavalent chromium (Cr(VI)) to non-toxic trivalent chromium (Cr(III)) in a continuous flow photoreactor. Additionally, Hou et al. [60] developed a facile one-pot synthesis strategy for constructing thiazole-linked COF-S and demonstrated its practical applicability by immobilizing it in a continuous flow system, where it achieved efficient degradation of organic pollutants under natural sunlight irradiation (Fig. 6b).

The rational design of continuous flow reactors integrated with COF-based catalysts shows significant promise for advancing energy chemistry and biochemistry applications. For instance, P. Biswal et al. [61] developed a single-site photocatalytic system based on a novel nitrogen/sulfur-containing TpDTz-COF-PS framework, which exhibits strong visible-light absorption and maintains structural stability during prolonged hydrogen evolution. Furthermore, the authors implemented an innovative continuous flow configuration that enables non-invasive, real-time monitoring of hydrogen production rates (Fig. 6c). This platform not only offers enhanced quantification accuracy but also provides unprecedented mechanistic insights that are challenging to obtain using conventional batch reactors. Additionally, a COF-based support material was utilized for the co-immobilization of enzymes and whole cells, facilitating synergistic catalysis between fructooligosaccharides and D-isomerase-expressing E. coli. When deployed in a column reactor packed with INU-NH_2_&E-NH_2_@NKCOF-141 (Fig. 6d), this system achieved a productivity of 161.28 g L^−1^ d^−1^ while retaining over 90% of its initial catalytic activity after 7 days of continuous operation [62].

Notably, advances in microreactor engineering and carrier design are systematically addressing the mass transfer limitations of COFs in continuous flow systems. The integration of COFs into continuous flow microreactors substantially enhances both reaction efficiency and catalyst stability. The “Put&Play Automated Microplatform (PPAM)” system developed by Deng et al. [63] achieved an 82% conversion rate of nitrobenzene in just 1 min of residence time by regulating microchannel size and catalyst coating method (PCC), with a TOF of up to 60 times that of batch reactions and an aniline yield of 8.8 g h^−1^. Computational fluid dynamics simulations showed that there were no “dead zones” commonly found in batch reactions in microchannels, significantly improving mass transfer efficiency.

In terms of enzyme catalysis, Feng et al. [64] constructed COF microcapsules (CALB@COF-MCs-SH). In a continuous flow system, a 97% esterification yield was achieved, and the catalytic efficiency (CE) was increased by 1.56 times compared to batch reactions after stable operation for 72 h. The microcapsule shell has size selectivity and can block the entry of large molecules such as proteases, significantly improving the operational stability of enzymes.

#### Continuous Flow Catalytic Reaction of COPs

**Fig. 7:**
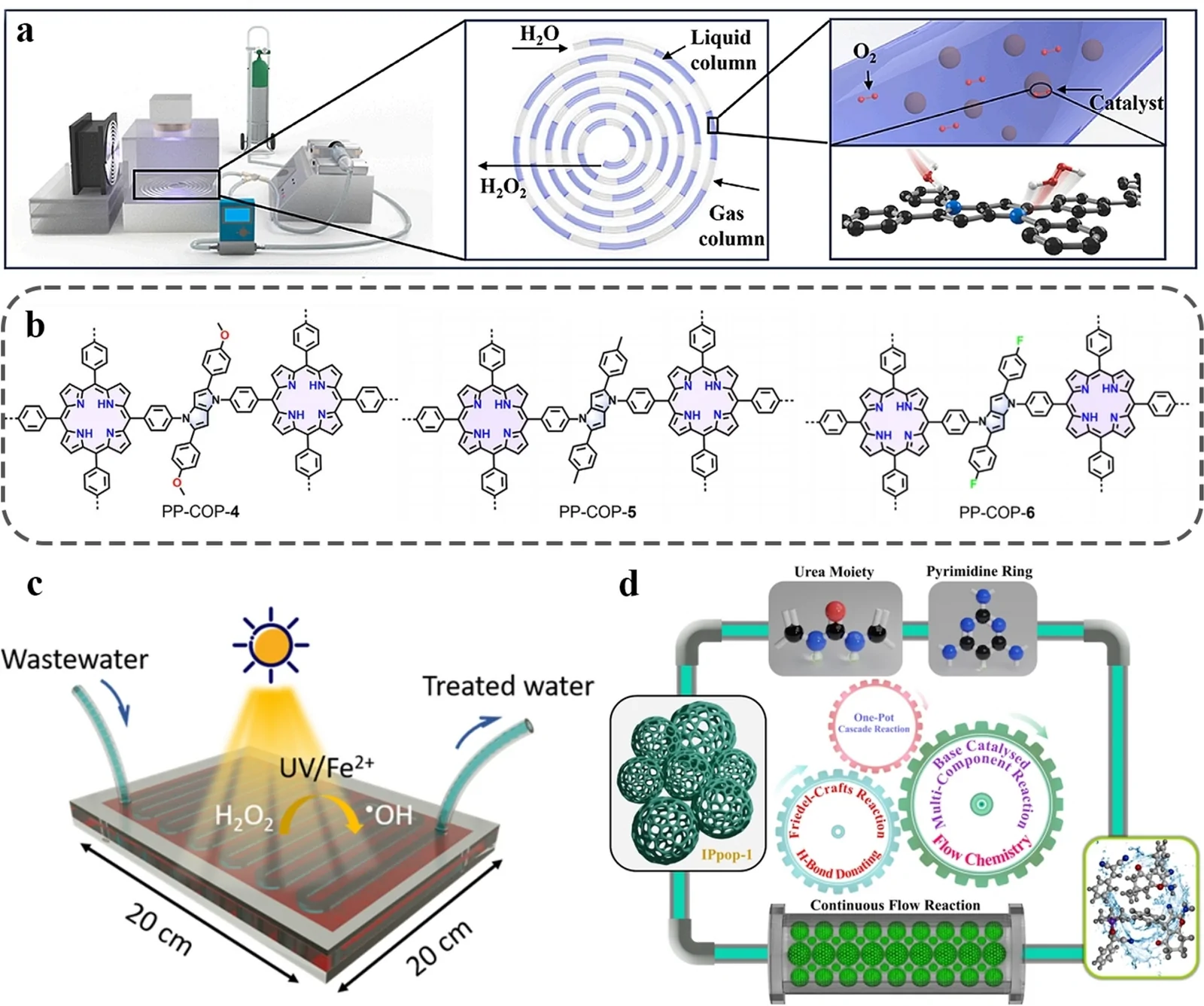
COPs for continuous flow catalysis. **a** Schematic representation of the microreactor platform and photocatalytic H_2_O_2_ reaction process [65]. Copyright 2024, Elsevier. **b** Structures of PP-COPs 4–6. [66]. Copyright 2025, Wily-VCH. **c** Photo-self-Fenton water treatment with panel reactor [67]. Copyright 2024, Wily-VCH. **d** Schematic illustration of multifunction catalytic sites, multifunctional activity, and multicomponent flow catalysis of by IPpop-1 [69]. Copyright 2024, American Chemistry Society

COPs, a class of porous materials characterized by tunable pore structures, high specific surface areas, and exceptional stability, have recently demonstrated considerable promise in the photocatalytic synthesis of H_2_O_2_. Conventional photocatalytic systems are often constrained by the limited oxygen solubility at the catalyst surface and slow mass transfer kinetics, which impede further enhancement of H_2_O_2_ yield. To overcome these limitations, Li et al. [65] designed and synthesized a series of COPs based on a pyrrolo[3,2-*b*]pyrrole structural motif. Among them, COP-2 achieved an H_2_O_2_ production rate of 5446 μmol g^−1^ h^−1^ in the absence of sacrificial agents, a performance attributed to its favorable hydrophilicity, high oxygen adsorption capacity, and efficient charge separation. Furthermore, by integrating COP-2 into a spiral tubular photoreactor, the authors constructed a gas–liquid–solid three-phase reaction system, which boosted the H_2_O_2_ yield to 20,285 μmol g^−1^ h^−1^ under continuous flow conditions (Fig. 7a). This result underscores the significant advantages of microreactors in improving mass transfer and enabling continuous production. In a further development, Fu et al. [66] proposed a “self-marketing & cooperation” strategy, incorporating two independent oxygen reduction active centers of pyrrolo[3,2-*b*]pyrrole and porphyrin units into a single polymeric framework to construct PP-COPs. Under light irradiation, this material simultaneously generates multiple reactive oxygen species, such as singlet oxygen (^1^O_2_) and superoxide radicals (·O_2_^−^), which act synergistically to promote H_2_O_2_ formation. The optimized material, PP-COP-4, attained a remarkable H_2_O_2_ production rate of 54,488 μmol g^−1^ h^−1^ in batch reactions. Moreover, when operated continuously in a plate microreactor for 9 h, it achieved a cumulative H_2_O_2_ concentration of 16.2 mM, significantly surpassing most reported metal-free photocatalysts (Fig. 7b). This study not only validates the efficacy of dual-active-site cooperative catalysis but also highlights the potential of continuous flow systems for achieving high-concentration, high-efficiency H_2_O_2_ synthesis.

With the development of research systems, the functional design of COP materials has evolved from solely producing hydrogen peroxide to integrated systems for synergistic pollutant degradation and resource utilization. Chu et al. [67] employed a multicomponent reaction (MCR) strategy to construct a sulfur-functionalized (C=S) polymer capable of achieving high H_2_O_2_ production (3132 μmol g^−1^ h^−1^) in pure water without external oxygen aeration (Fig. 7c). To demonstrate its applicability in situ wastewater treatment, the authors designed a panel reactor system (20 cm × 20 cm) for large-scale H_2_O_2_ generation. They observed that the photo-self-Fenton system utilizing in situ-generated H_2_O_2_ exhibited a 7.9-fold higher utilization efficiency compared to conventional photo-Fenton processes. Abolhasani et al. [68] reported a heterogeneous flow chemistry approach for the accelerated chemoselective hydrogenation of nitroarenes using a palladium catalyst supported on a poly(β-cyclodextrin) network. The developed packed-bed flow reactor enabled selective hydrogenation of a diverse range of nitroarenes with > 99% yield under ambient conditions and short residence times (1 min). Dam et al. [69] fabricated a porous urea-based network (IPpop-1) that functions as an efficient heterogeneous hydrogen-bond-donating (HBD) catalyst for the Friedel–Crafts alkylation of β-nitrostyrene and indole, yielding up to 99% under mild conditions in line with green chemistry principles (Fig. 7d). To enhance practical utility, the catalyst was shaped into spherical composite beads and successfully applied in continuous flow multicomponent Knoevenagel–Michael condensation without loss of activity. Ma et al. [70] synthesized iPAF-7, a framework functionalized with cationic imidazolium groups, which demonstrated excellent capability for gold recovery from electronic waste leachate. The adsorbed gold was subsequently reduced to form gold nanoparticles immobilized within the polymer, yielding a composite catalyst denoted as Au@iPAF-7. Additionally, the incorporation of iPAF-7 into an aerogel monolith enabled continuous flow catalytic reduction of nitroarenes.

#### Advantages and Disadvantages of Continuous Flow Catalysis of COFs/COPs

##### Key evaluation of the advantages and disadvantages of COF/COPs materials in continuous flow catalytic reactions

*Advantages*: (I) Highly ordered pore structure: COF/COPs have regular and adjustable pores, providing ideal high-speed channels for the transport of reactants and products, effectively reducing mass transfer resistance and improving reaction efficiency. (II) Extremely high specific surface area and abundant active sites: The huge specific surface area enables highly dense distribution of catalytic active sites, thereby providing high catalytic activity. (III) Excellent structural designability: Its modular synthesis allows for precise design and control of the chemical environment and size of pores at the molecular level, enabling customization of advanced catalytic functions such as chiral catalysis and specific substrate size selection. (IV) Good stability: Compared to MOFs, most COF/COPs are connected by strong covalent bonds and have excellent chemical and thermal stability. They can maintain structural integrity under harsh reaction conditions and various solvents, making them suitable for long-term operation (Table 3). (V) Reduce catalyst loss: By taking COF/COPs into integral columns or fixed beds, heterogeneous catalysts can be firmly fixed in the reactor, greatly reducing the leaching and loss of catalysts in the flowing system, ensuring the purity of the reaction system and the long-term service life of the catalyst.

**Table 3:** Lifetime under different pH levels and temperatures of COF/COPs materials

COF/COPs Materials	pH Stability	Temperature stability	Refs
COF-BD2	Stable over a broad pH range of 3–11, maintaining high-efficiency H_2_O_2_ production in aqueous solutions at different pH levels	Excellent thermal stability, withstanding temperatures up to 400 °C	[45]
TpBpy	Stable in ethanol, DMF, pH = 2 (HCl), and pH = 12 (NaOH). A mass loss of 10% is observed at pH = 12, while in other solvents, the mass loss is < 3%	Stable above 400 °C under nitrogen atmosphere, with a mass loss < 5% within the effective temperature range	[56]
dDAAQ-TFP	Capable of efficiently catalyzing reactions over a wide pH range of 3–10	No significant thermal decomposition below 600 °C	[59]
PP-COP-4	Stable within the pH range of 1–9, with optimal catalytic activity observed at pH = 5. Maintains high catalytic efficiency in various water sources (seawater, lake water, tap water)	Thermally stable up to 250 °C, with no significant structural decomposition below this temperature	[66]
iPAF-7	Stable in aqua regia systems (pH < 1) for adsorption of gold ions	Thermally stable up to 240 °C, with structural integrity maintained below this temperature	[70]

*Disadvantages*: (I) Challenges in processing and shaping: COFs/COPs are typically obtained as fine powders, and their processing into macroscopic structures such as monoliths, beads, or thin films suitable for fixed-bed continuous flow reactors remains a significant challenge. These shaping processes are often complex and risk compromising material porosity and structural integrity. (II) Persistent mass transfer limitations: Although COFs possess ordered pore structures at the molecular level, macroscopic mass transfer can become a rate-limiting factor when particles are densely packed. Diffusion through interparticle voids and along long, tortuous pore pathways may hinder overall reaction efficiency. (III) Long-term stability and regeneration issues: Under practical continuous flow conditions, COFs/COPs are susceptible to gradual deactivation caused by mechanical abrasion, chemical degradation, or irreversible active-site poisoning. Regeneration of spent COF/COP materials is often difficult and inefficient, posing a barrier to sustained application. (IV) Cost and scalability concerns: The synthesis of high-purity monomers, fabrication of COFs, and subsequent macroscopic shaping processes tend to be costly and technically demanding. These factors currently impede large-scale, economical production and limit broader industrial adoption. (V) Limitations in catalytic active sites: Active sites introduced via post-synthetic modification may suffer from heterogeneous distribution or limited accessibility. Meanwhile, sites directly constructed from monomer units often exhibit inferior activity and diversity compared to conventional molecular catalysts.

### Cages for Continuous Flow Catalysis

As a class of supramolecular assemblies with well-defined cavity architectures, cages can mimic enzymatic active sites, enabling efficient and highly selective catalytic conversions within confined spaces. However, conventional homogeneous cage systems suffer from limitations such as difficult recovery, limited structural stability, and challenges in continuous operation, which hinder their practical deployment. To overcome these drawbacks, researchers have been pursuing heterogeneous strategies for cage immobilization and their integration into continuous flow catalytic systems. Such approaches aim to achieve robust, recyclable, and stable catalytic processes, thereby advancing the practical application of molecular cages in fine chemical and pharmaceutical synthesis.

**Fig. 8:**
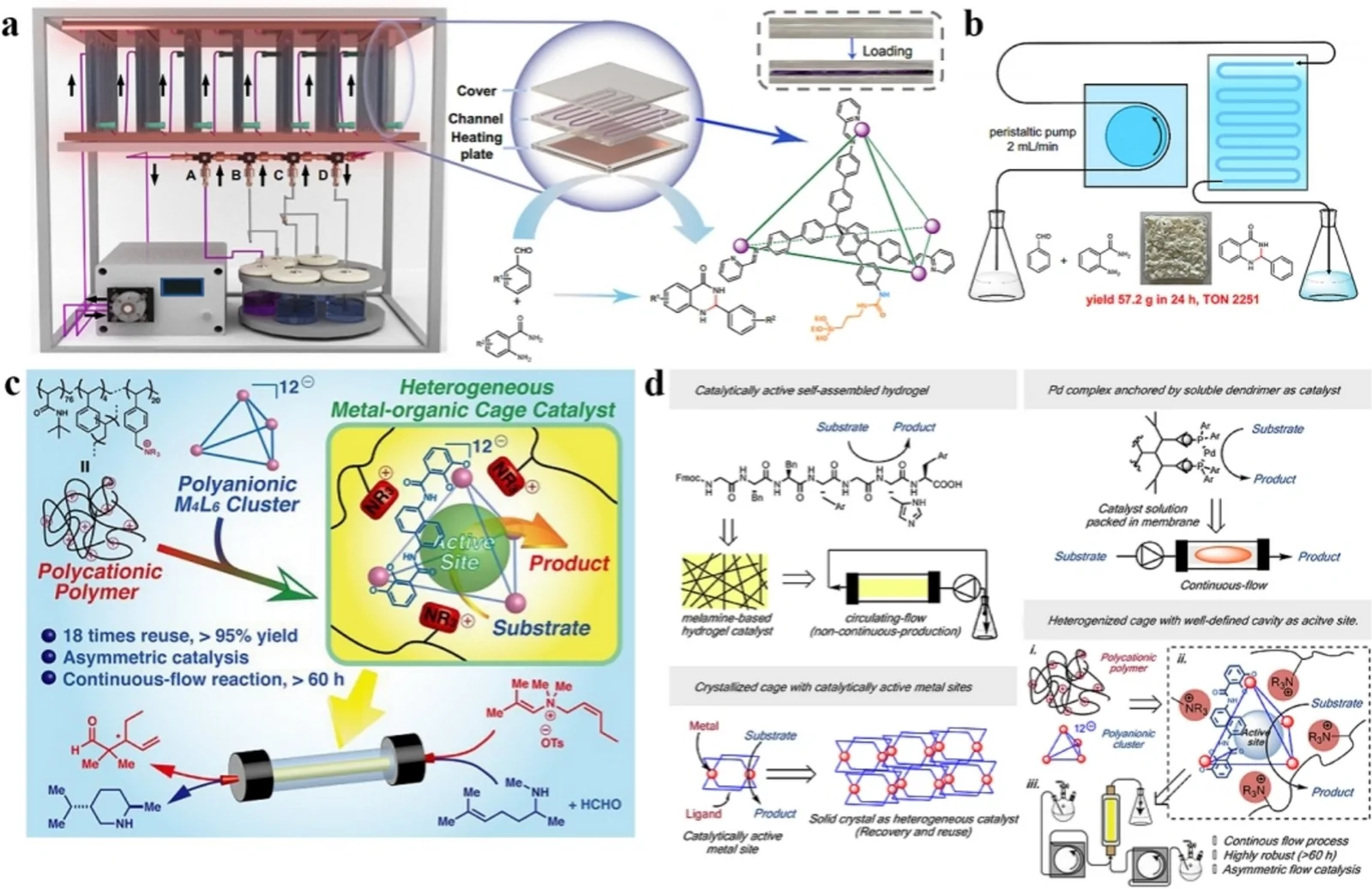
Cages for continuous flow catalysis. **a** Schematic of the CLSC model display. **b** Schematic of the amplified continuous flow experimental system [71]. Copyright 2024, Springer Nature. **c** Heterogeneous supramolecular catalysis through immobilization of anionic M_4_L_6_ assemblies on cationic polymers. **d** Heterogeneous supramolecular catalysts and flow systems [73]. Copyright 2020, American Chemistry Society

In the context of heterogeneous strategies, covalent immobilization has garnered significant attention due to its exceptional stability. Li et al. [71] precisely engineered the self-assembly process to construct two amine-functionalized Fe_4_L_4_ tetrahedral metal–organic cages (MOCs), denoted as cages 1 and 2, with distinct cavity sizes. Employing a post-synthetic modification method, the authors functionalized the uncoordinated amino groups on the cage surfaces with 3-isocyanatopropyltriethoxysilane (IPTS), successfully grafting triethoxysilane chains to form the derived materials Si-1 and Si-2. These functionalized MOCs were then covalently anchored onto the inner walls of polydimethylsiloxane (PDMS) microchannels, establishing a highly integrated “Catalyst Loading and Substrate Conversion” (CLSC) automated microfluidic catalytic system (Fig. 8a, b). This system demonstrated outstanding performance in the cyclization reaction of ortho-aminobenzamide with aldehydes, achieving high conversion and broad substrate adaptability. Moreover, it maintained stable operation for over 20 cycles under continuous flow conditions without significant loss of activity. The remarkable catalytic behavior is attributed to the efficient substrate confinement within the MOC cavities, enhanced mass transfer enabled by the microreactor architecture, and the structural robustness imparted by covalent bonding. This work represents the first example of covalent immobilization and continuous flow catalysis of functional MOCs within a microfluidic environment, offering a valuable reference for the future development of automated and modular synthesis systems. Similarly, Ye et al. [72] introduced the reduction product of imine-based porous organic cage CC1 (RCC1) as a novel crosslinker in fabrication of polymeric monolith by ring-opening polymerization with ethylene glycol diglycidyl ether (EGDGE). Owing to the amine-rich polymeric framework, the resulting poly(RCC1-co-EGDGE) monoliths functioned as dual-purpose reductants and stabilizers, enabling the in situ generation of gold nanoparticles (AuNPs) on their surfaces through the spontaneous reduction of Au^3^⁺ to Au⁰. The AuNP-decorated poly(RCC1-co-EGDGE) monolith was subsequently utilized as an integrated catalytic microreactor for the reduction of 8-nitroquinoline to 8-aminoquinoline. Complete consumption of the reactant was observed in the effluent, and the target product was obtained in high yield, underscoring the considerable potential of RCC1-derived monolithic architectures in continuous flow catalytic applications.

In contrast to covalent immobilization strategies, non-covalent interactions (such as electrostatic forces, host–guest interaction, and hydrogen bonding) offer a dynamically tunable alternative pathway for the heterogenization of cages. These approaches are generally facile to operate and can preserve or even enhance the inherent flexibility and responsiveness of the molecular cages. Miyamura et al. [73] developed a heterogeneous catalytic system based on electrostatic interactions. They immobilized highly electronegative (12e^−^) Ga_4_L_6_ chiral supramolecular cages onto cross-linked polymers bearing cationic functional groups via multivalent electrostatic attraction, constructing a novel class of heterogeneous supramolecular catalysts (Fig. 8c, d). These catalysts not only preserve the cavity-mediated catalytic behavior characteristic of their homogeneous counterparts in reactions such as aza-Prins cyclization and aza-Cope rearrangement, but also exhibit superior catalytic activity and significantly enhanced operational stability. Studies revealed that the ammonium cations in the polymer support not only serve to anchor the cages but also form an outer protective layer that modulates the conformational dynamics and cavity accessibility of the MOCs, thereby influencing both catalytic performance and enantioselectivity. Notably, in a continuous flow system, the heterogeneous catalyst displays reversible “inhibition-reactivation” behavior, which can be regulated by the addition of ammonium salts as allosteric guests, mimicking the allosteric regulation mechanism observed in enzymatic catalysis. The immobilized chiral cages maintain high enantioselectivity even after multiple recycling and under continuous flow conditions, highlighting the stabilizing role of the heterogeneous support in preserving the chiral configuration of MOCs. Yang et al. [74] developed a simple and efficient supramolecular coordination cages (SCCs) template strategy to synthesize ultrasmall noble metal nanocatalysts (UNMNs) with controllable size and size distribution. A series of SCCs, including M_2_L_4_, M_4_L_2_, M_6_L_4_, and M_12_L_24_, with well-defined sizes and shapes as well as different numbers of Pd ions were designed and synthesized as templates. Subsequently, the corresponding Pd nanocatalysts of M_2_@CMC, M_4_@CMC, M_6_@CMC, and M_12_@CMC were prepared by an impregnation–reduction method on the support of carboxymethylcellulose (CMC) hydrogels. Furthermore, the as-prepared Pd nanocatalyst could serve as highly efficient polychlorinated biphenyls (PCBs) degrader both in the stirred vessel and continuous flow reactor because of its excellent catalytic efficiency in reductive dehalogenation reaction under the mild conditions.

Moreover, the application of cages as homogeneous catalysts in continuous flow systems has garnered increasing attention. Xu et al. [75] demonstrated a simple but highly efficient means of supramolecular self-assembly of metallacages in microdroplets by using a continuous flow microfluidic approach. The high efficiency and versatility of this strategy were demonstrated by the highly efficient self-assembly of five different metallacages in microdroplets in a few min with nearly quantitative yields. Furthermore, the catalytic performance of these metallacages was evaluated under microdroplet conditions, reinforcing the superiority and broad applicability of the strategy. Kinetic and thermodynamic analyses revealed a substantial enhancement in catalytic reaction efficiency relative to conventional batch reactors. Notably, both the catalytic turnover number (K_cat_) and the rate constant (K) exhibited an inverse correlation with microdroplet diameter, a phenomenon attributable to pronounced volume confinement effects.

In summary, the heterogenization of cages through covalent or non-covalent strategies and their integration into continuous flow reaction systems has become a key pathway for expanding their practical applications. Both strategies have their advantages: Covalent immobilization endows the system with higher stability and is suitable for harsh reaction conditions, while non-covalent fixation is more convenient to operate and facilitates the regulation of the microenvironment and dynamic behavior of cages. The high mass and heat transfer efficiency, precise reaction control, and ease of amplification inherent in continuous flow technology complement the limited catalytic ability of cages, jointly enhancing the efficiency and sustainability of catalytic processes. Furthermore, the catalytic application of cages in homogeneous continuous flow systems offers an innovative pathway for developing highly efficient catalytic processes.

## Inorganic Materials for Continuous Flow Catalysis

Inorganic porous materials, particularly zeolites and mesoporous silica, play a pivotal role in continuous flow catalysis. While both belong to the family of silicon-based porous materials, their distinct structural characteristics and functional roles enable them to form a complementary and synergistic system in practical applications. Zeolites possess a well-ordered microporous structure (with pore sizes < 2 nm), tunable acidity, and excellent thermal stability, making them particularly suitable for shape-selective catalysis of small molecules and transformation processes requiring high stability [76–78]. However, their narrow micropores may restrict the diffusion of larger reactants or products, leading to mass transfer limitations. In contrast, mesoporous silica features larger pore sizes (2–50 nm), high specific surface area, and a flexibly tunable pore structure. Although it generally lacks intrinsic catalytic activity, it serves as an excellent support material, effectively enhancing mass transfer and enabling the highly dispersed loading and precise assembly of active sites [79, 80]. In continuous flow catalytic systems, zeolites are often employed as intrinsically active catalysts, directly participating in acid-catalyzed or metal-mediated cooperative reactions. Mesoporous silica, on the other hand, is primarily utilized as a functionalized support, improving mass transfer efficiency through its open pore channels and facilitating the synergistic catalysis of multiple active centers. This microporous-to-mesoporous material framework addresses the dual demands of continuous flow catalysis for both catalytic activity and mass transfer performance, collectively establishing a multi-level catalytic strategy capable of handling molecules of varying sizes and complex reaction networks.

### Zeolites for Continuous Flow Catalysis

Zeolites characterized by their well-defined microporous structures, tunable acidity, and exceptional thermal stability, hold a significant position in the field of catalysis. In recent years, the application of zeolites in continuous flow catalytic reactions has garnered widespread attention, particularly demonstrating unique advantages in energy conversion and environmental catalysis. Zeolites not only serve as conventional fixed-bed catalysts but can also be structurally designed to achieve effective encapsulation and stabilization of metal nanoparticles, significantly enhancing catalytic performance and longevity.

**Fig. 9:**
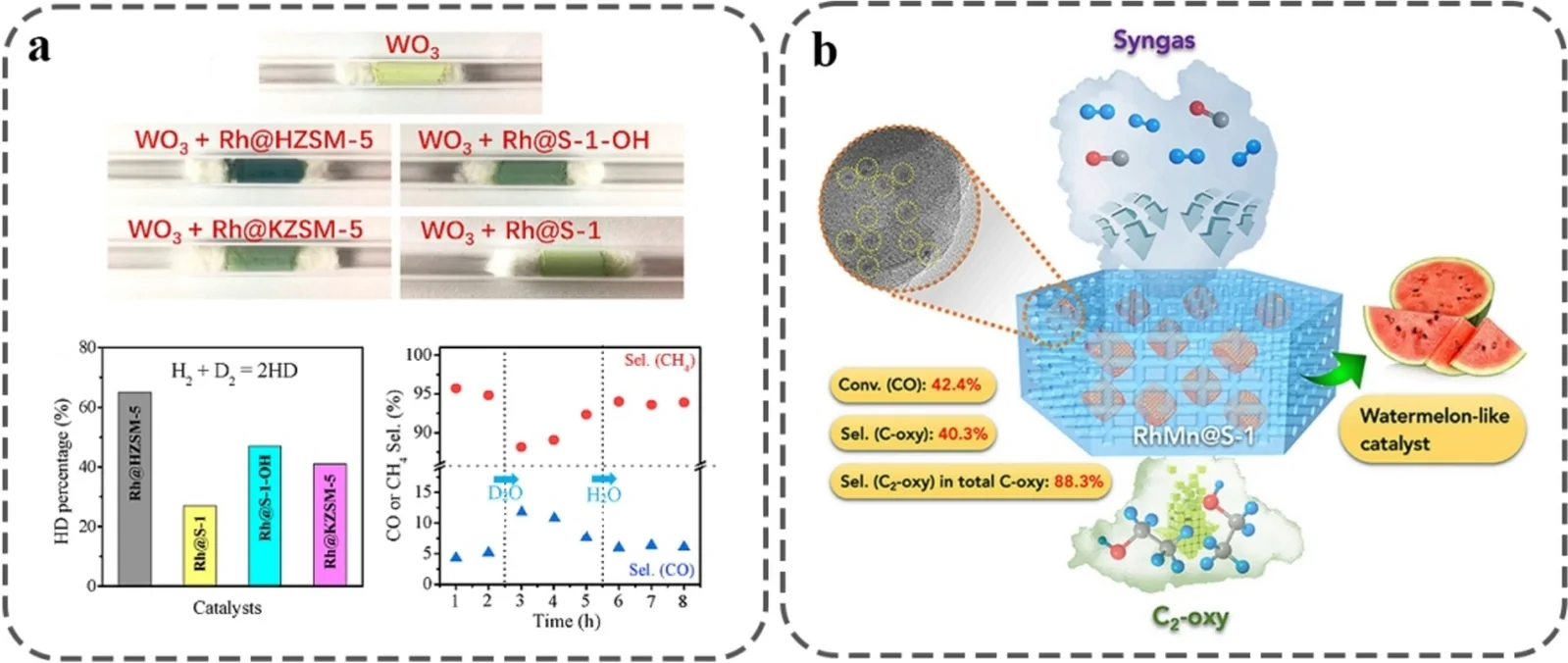
Zeolites for continuous flow catalysis. **a** Photographs of samples made with 1 g of WO_3_ mixed with 0.02 g of various catalysts after treatment with H_2_ at 30 °C for 10 min [81]. Copyright 2019, American Chemistry Society. **b** Direct conversion of syngas to ethanol within zeolite crystals [83]. Copyright 2020, Elsevier

Wang et al. [81] achieved precise control over product selectivity in the CO_2_ hydrogenation reaction within a continuous flow reactor by encapsulating Rh nanoparticles within an MFI-type zeolite framework (Rh@zeolites) (Fig. 9a). The catalytic tests were performed in an upright stainless steel fixed-bed continuous flow reactor, where the zeolite catalyst was mixed with quartz sand for packing. The reactions were conducted under a pressure of 1 MPa and an H_2_/CO_2_ ratio of 3. The continuous flow operation ensured sustained contact between reactants and the catalyst, facilitating the acquisition of steady-state data and precise analysis of reaction pathways. Experimental results revealed that Rh@S-1, at a CO_2_ conversion of 51.6% and 500 °C, exhibited high CO selectivity of 79.8%. In contrast, Rh@HZSM-5, achieving a higher conversion of 68.2%, primarily produced methane with a selectivity of 98.2%. This marked difference underscores the regulatory role of the nanochannels’ microenvironment within the zeolite on the adsorption and diffusion behaviors of reaction intermediates, the hydrophobicity and weak adsorbility of S-1 promote rapid CO desorption, inhibiting its further hydrogenation to methane, whereas the acid sites in HZSM-5 enhance CO adsorption and hydrogen spillover, thereby promoting the methanation reaction.

Zhang et al. [82] introduced a fabrication strategy for metal@zeolite composite materials based on a “seed-directed growth” approach. This method successfully encapsulated metal nanoparticles (e.g., Pt, Pd, Rh, Ag) within the internal structure of zeolite crystals, yielding metal@zeolite catalysts with precisely controlled sizes (0.8–3.6 nm). The strategy involves embedding pre-synthesized metal-loaded zeolite seeds into a silicoaluminate gel, followed by hydrothermal crystallization to form a complete zeolite shell, thereby firmly anchoring the metal nanoparticles inside the zeolite framework. This approach circumvents issues common in traditional impregnation methods, such as metal particle sintering and migration. The results demonstrated that Pt@Beta maintained platinum nanoparticle sizes in the range of 0.8–3.2 nm even after calcination in air at 600 °C for 240 min, exhibiting exceptional resistance to sintering.

Wang et al. [83] employed a zeolite immobilization strategy, encapsulating RhMn-based active nanoparticles within the crystals of pure silica zeolite silicalite-1 (S-1) via a solvent-free zeolite crystallization method, thereby constructing a core–shell structured catalyst (Fig. 9b). The evaluation of this catalyst in the direct ethanol synthesis from syngas, conducted in a continuous flow fixed-bed reactor, revealed excellent catalytic performance. It attained a CO conversion of 42.4% with a concomitant selectivity to C-containing oxygenates of 40.3%, notably, C_2_-oxygenates accounted for a high proportion (88.3%) among the total oxygenates, resulting in an ethanol production rate of 80.6 mol h^−1^. These metrics are significantly superior to those of traditional supported Rh-based catalysts, highlighting the advantages of the zeolite encapsulation structure in enhancing catalytic performance. These findings highlight the multifunctional role of zeolites in continuous flow catalytic systems, encompassing “directional regulation”, “stable encapsulation”, and “performance enhancement”, thereby providing a crucial paradigm for the rational design of catalysts in fields such as energy conversion and environmental catalysis.

### Mesoporous Silica for Continuous Flow Catalysis

Mesoporous silica materials, renowned for their high specific surface area, tunable pore structures, and good chemical stability, exhibit broad application prospects in catalysis. In recent years, continuous flow catalytic technology has gradually become a research hotspot in catalytic materials due to its advantages of high efficiency, controllability, and ease of scale-up. The integration of mesoporous silica materials with continuous flow reactors not only enhances the efficiency and selectivity of catalytic reactions but also paves new pathways for green and sustainable chemical synthesis.

In continuous flow reactors, mesoporous silica is frequently utilized as a catalyst support, enabling high dispersion and stable immobilization of active components through its ordered pore channels. Yepez et al. [84] developed a continuous flow synthesis method for depositing Fe_2_O_3_ nanoparticles onto AlZn-SBA-15 mesoporous silica. The reactor setup consisted of a stainless steel packed bed filled with the mesoporous silica support. A metal precursor solution was passed through the bed at different flow rates (0.1–2 mL min^−1^) under mild heating (100 °C), achieving in situ deposition of nanoparticles. This system yielded uniformly sized and well-dispersed Fe_2_O_3_ nanoparticles within very short residence times, while the material maintained a high specific surface area exceeding 700 m^2^ g^−1^ and a pore size of approximately 6 nm, indicating that Fe species were primarily deposited on the external surface without causing pore blockage. This material exhibited excellent catalytic performance in the oxidation of benzyl alcohol and benzylation of toluene, achieving conversions up to 99%, with catalytic activity surpassing that of catalysts prepared by traditional impregnation, microwave-assisted, and mechanochemical ball-milling methods.

**Fig. 10:**
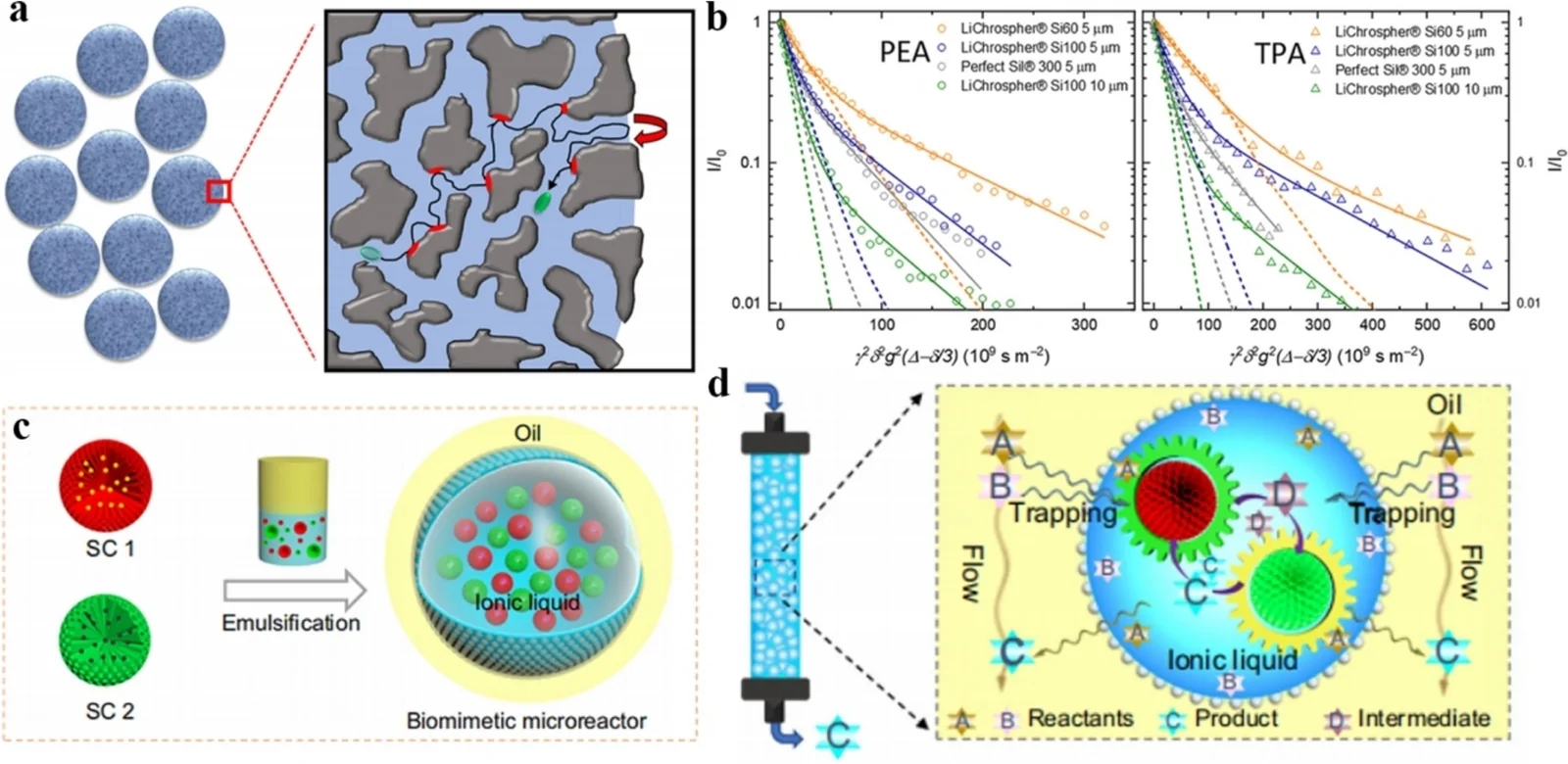
Mesoporous silica for continuous flow catalysis. **a** Sketch of the diffusion pathway of a molecule in a porous silica particle. **b** PFG NMR analysis: Diffusion attenuation curves of PEA and TPA in LiChrospher Si60 5 μm (orange), LiChrospher Si100 5 μm (blue), LiChrospher Si100 10 μm (green), and Perfect Sil 300 5 μm with a biexponential fit (solid lines) and a fit of the initial signal attenuation with the model of restricted diffusion in a sphere according to Balinov et al. [85]. Copyright 2025, American Chemistry Society. **c** Co-localization of two distinct catalytically active subcompartments (SCs) within a single droplet of ionic liquid-in-oil Pickering emulsion generating a biomimetic microreactor. **d** Column reactor packed with the biomimetic microreactors for continuous flow cascade reactions [89]. Copyright 2022, Springer Nature

In the study of mesoporous silica support materials, Trommer et al. [85] systematically evaluated the performance of spherical silica particles with different mesoporous diameters of 6, 10, and 30 nm in the esterification reaction catalyzed by immobilized organic catalyst DMAP (4-(dimethylamino)pyridine). The study analyzed mass transfer limitations through nitrogen physical adsorption, pulsed field gradient nuclear magnetic resonance (PFG NMR), and Weisz–Prater criterion parameters (Φ_WP_) (Fig. 10a). In batch reactor, the conversion rate of α-tocopherol (TP) in 6 nm mesoporous materials is only about 20% due to poor pore connectivity and strong liquid surface interactions, while the conversion rates in 10 and 30 nm materials reach 40% and 60%, respectively. In contrast, in the continuous flow reactor, the conversion rate of TP was significantly improved by 10 and 30 nm materials, especially at a low flow rate of 0.05 mL min^−1^ (Fig. 10b). The conversion rate of 30 nm material was close to the intermittent level, but the TOF value was higher. The self-diffusion coefficient determined by PFG NMR showed that the effective diffusion coefficient (Deff) of TP in the 30 nm material was 1.2 × 10^–10^ m^2^ s^−1^, much higher than that in the 6 nm material (0.5 × 10^–10^ m^2^ s^−1^), confirming the critical influence of mesoporous size on mass transfer. Weisz–Prater analysis further showed that the Φ_WP_ value of the 6 nm material was greater than 0.3, indicating that the reaction was controlled by mass transfer, while the Φ_WP_ values of the 10 and 30 nm materials were lower than 0.3, indicating that reaction kinetics dominated. These results highlight the advantages of continuous flow reactors in overcoming mass transfer limitations, especially when dealing with large molecule substrates, where flow conditions can enhance substrate to active-site contact and reduce product retention.

Pickering emulsion-templated synthesis offers a versatile route to architecturally controlled porous silica materials for continuous flow catalysis. By utilizing emulsion droplets as dynamic templates, this approach enables precise fabrication of hierarchical structures, such as hollow, yolk–shell, or interconnected porous networks, with tunable pore geometry and surface functionality [86–88]. These tailored materials enhance mass transport and active-site accessibility in flow reactors, while allowing in situ integration of catalytic species (e.g., metal nanoparticles or functional groups). Demonstrated in hydrogenation, oxidation, and cascade reactions, such structured catalysts combine high activity with improved stability under continuous operation. Zhang et al. [89] constructed a biomimetic microreactor system based on Pickering emulsions for continuous flow cascade catalysis. In this system, mesoporous silica nanoparticles (MSNs) served as catalytic subcompartments, loaded with Ti(Salen) complex and lipase CALB, respectively. These were co-localized within ionic liquid microdroplets via an emulsification process, forming microreactors resembling cellular structures. These microreactors were packed into a column reactor, and reactants in the oil phase flowed continuously through, contacting the catalysts within the microreactors (Fig. 10c). This setup not only realized the spatial isolation and synergistic action of different catalysts but also significantly enhanced the local concentration of reactants leveraging the enrichment effect of the ionic liquid, thereby substantially boosting catalytic efficiency (Fig. 10d). In the cascade synthesis of chiral cyanohydrins and chiral esters, this system achieved up to a 420-fold enhancement in catalytic efficiency and 99% enantioselectivity, maintaining stability over 240 h of continuous operation. Yang et al. [90] prepared multicompartmentalized liquid-containing microreactors (MLMs) through stepwise Pickering emulsification and interfacial cross-linking, successfully spatially isolating enzymes and metal catalysts within distinct subcompartments. These microreactors achieved the dynamic kinetic resolution of alcohols under continuous flow conditions, maintaining stable operation for over 2000 h with 99% enantioselectivity. Furthermore, they utilized a Pickering emulsion interface-directed synthesis strategy to construct enzyme microreactors with MOF shells. The MOF layer endowed the reactors with size-selective permeability, enabling stable operation for over 1000 h in continuous flow biocatalytic systems while maintaining enantioselectivity comparable to that of free enzymes. These two studies, through the Pickering emulsion templating method, provide novel biomimetic microreactor design paradigms for continuous flow biocatalysis from the perspectives of multicompartmental isolation and size-selective sieving, respectively [91].

## Other Materials for Continuous Flow Catalysis

### Polymeric Carbon Nitride (PCN) for Continuous Flow Catalysis

PCN is a non-metallic semiconductor material with excellent light absorption properties, controllable band structure, and good chemical stability. In recent years, it has shown broad application prospects in the field of heterogeneous photocatalysis. Compared with traditional batch reactors, PCN is widely used in continuous flow catalytic systems to construct efficient, stable, and scalable photocatalytic reaction platforms due to its high specific surface area, controllable pore structure, and ease of immobilization.

**Fig. 11:**
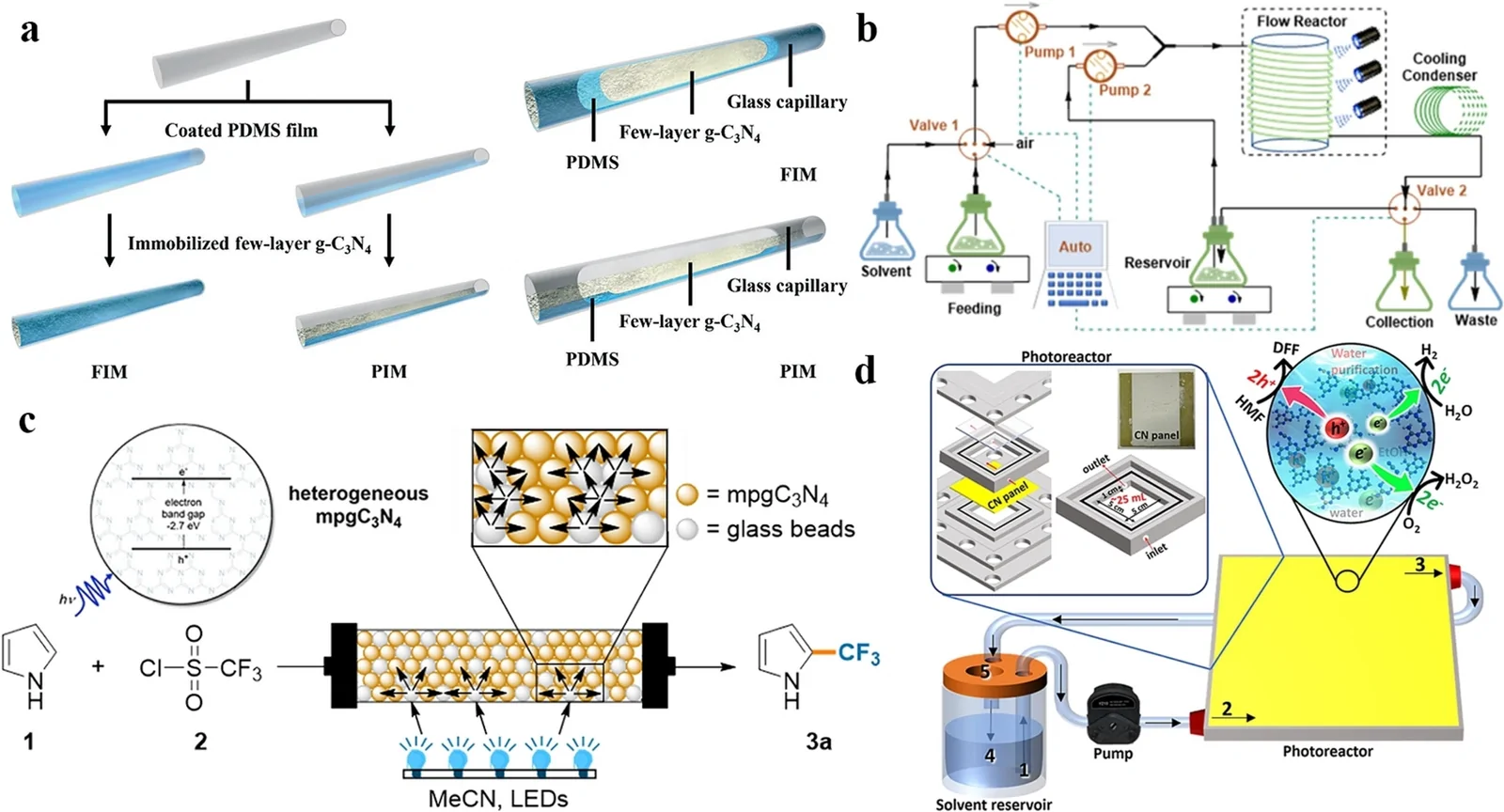
PCNs for continuous flow catalysis. **a** Simple procedure to fabricate the FIM and PIM [92]. Copyright 2022, Royal Society of Chemistry. **b** Schematic illustration of the semicontinuous circulation flow synthesis with automated feeding and collection [93]. Copyright 2024, American Chemistry Society. **c** General scheme for the continuous flow trifluoromethylation of pyrrole [94]. Copyright 2023, American Chemistry Society. **d** Schematic diagram of the experimental setup: The reactant solution is continuously pumped from a solvent reservoir (1) using a peristaltic pump through the inlet (2) and, after flowing in the photoreactor over the photocatalyst panel (yellow), flows out through the outlet (3) back into the solvent reservoir (4). The solvent reservoir is equipped with a rubber septum (5) for gas purging and liquid sampling [95]. Copyright 2024, American Chemistry Society

In continuous flow reaction systems, the immobilization of catalysts is crucial for enhancing reaction efficiency and operational stability. Although traditional slurry reactors can achieve high dispersion, they suffer from challenges such as difficult catalyst recovery and non-uniform light utilization. Huang et al. [92] developed a microreactor based on glass capillaries, in which a polydimethylsiloxane (PDMS) layer was controllably coated on the inner wall to immobilize g-C_3_N_4_, successfully constructing a fully immobilized microreactor (FIM) and a partially immobilized microreactor (PIM) (Fig. 11a). The study demonstrated that under continuous flow conditions, the FIM achieved a NADH regeneration rate of 82.20% within 40 min, significantly higher than the 56.43% observed under static conditions. Although the PIM exhibited a lower overall NADH regeneration rate (48.15%, 40 min), it demonstrated superior efficiency per unit coating angle, with values of 0.437 mM/° (flow condition) and 0.427 mM/° (static condition), which were 1.91 and 2.72 times higher than those of the FIM, respectively. This indicates that the partial immobilization strategy offers unique advantages in terms of light and mass transfer. Furthermore, this continuous flow reactor system exhibited outstanding performance in enzymatic catalysis. Using glutamate dehydrogenase (GDH) as a model enzyme, the photoregeneration of NADH was employed to catalyze the conversion of α-ketoglutarate to L-glutamate, achieving a conversion rate as high as 99.92% within 30 min. This fully demonstrates the potential of PCN as an efficient photocatalyst in continuous flow photobiocatalytic systems. Similarly, Liu et al. [93] developed a high-speed recirculating flow reactor, in which the reaction mixture (containing substrate, solvent, and solid catalyst) was pumped from a reservoir, passed at high velocity through an irradiated tubular reactor, and then returned to the same reservoir (Fig. 11b). This process was continuously repeated until reaction completion. By increasing the flow rate, issues such as sedimentation and clogging of solid catalysts were effectively mitigated, enabling the scale-up of C-N and C-S coupling reactions, as well as trifluoromethylation, from hundreds of grams to kilogram scale. Moreover, the PCN photocatalyst could be reused more than 10 times without significant deactivation. This study highlights the ability of PCN to maintain good dispersion and catalytic activity under high-flow conditions, providing a feasible strategy for handling flow reactions involving solid catalyst systems.

Sivo et al. [94] systematically investigated the catalytic performance of nanostructured carbon nitride materials in the trifluoromethylation of aromatic hydrocarbons, with a focus on the effects of structural properties, specific surface area, and bandgap modulation on reaction efficiency. In this study, three types of carbon nitride materials with different structures of graphitic carbon nitride (g-C_3_N_4_), nanosheet carbon nitride (n-C_3_N_4_), and mesoporous graphitic carbon nitride (mpg-C_3_N_4_) were synthesized (Fig. 11c). Specific surface area measurements revealed that mpg-C_3_N_4_ possessed the highest specific surface area (157 m^2^ g^−1^), significantly exceeding those of g-C_3_N_4_ (5 m^2^ g^−1^) and n–C_3_N_4_ (72 m^2^ g^−1^). This structural advantage considerably enhanced its performance in the photocatalytic trifluoromethylation reaction, achieving a target product yield of 80%, whereas the other two materials yielded less than 10%. In a continuous flow reaction system, a packed-bed photoreactor was designed, utilizing a transparent FEP tube loaded with mpg-C_3_N_4_, K_2_HPO_4_, and glass beads (2.5 ωt%). Under blue light irradiation (*λ* = 457 nm), an optimal residence time of 20 min resulted in a yield of 77%. Compared to batch reactions, the continuous flow system not only enabled efficient catalyst recovery and reuse but also maintained stable activity over 5 h of continuous operation, equivalent to 15 batch reaction cycles. Scale-up experiments (5 mmol scale) further validated the feasibility of this process, achieving a yield of 62% and a production rate of 0.62 mmol h^−1^, significantly higher than the 0.32 mmol h^−1^ observed in batch reactions.

Beyond applications in organic synthesis, PCN has also been utilized in the construction of photocatalytic panels with potential for practical production. Battula et al. [95] reported a binder-free PCN panel capable of efficient hydrogen peroxide synthesis and oxidation of biomass-derived HMF under continuous flow conditions, demonstrating excellent light absorption and interfacial reaction performance. The CN panel was fabricated on a glass substrate via a “sandwich” thermal polymerization method, featuring a uniform porous structure and controllable thickness (Fig. 11d). Strong chemical bonding with the substrate ensured structural stability and resistance to reactive oxygen species (ROS) corrosion under continuous flow conditions. In a custom-designed continuous panel-flow reactor, the CN panel demonstrated outstanding multifunctional photocatalytic performance. Specifically, a thick-layer CN panel achieved a H_2_O_2_ production rate of up to 117 μmol h^−1^ g^−1^, indicating its potential for industrial-scale H_2_O_2_ synthesis. In the oxidation of 5-hydroxymethylfurfural (HMF) to 2,5-diformylfuran (DFF), an HMF conversion rate of 1185 μmol h^−1^ g^−1^ was attained with a DFF yield of 13%. With the assistance of a Pt cocatalyst, hydrogen evolution reached 5.84 μmol over a 4-h period. Additionally, aqueous-phase degradation of the organic dye rhodamine B achieved 58% removal after 110 min of continuous operation. The HMF conversion rate and reaction selectivity observed in this work represent state-of-the-art performance among continuous flow photocatalytic systems based on C_3_N_4_ materials. These studies have demonstrated that the synergistic integration of structural optimization and continuous flow engineering is pivotal for advancing the photocatalytic performance of PCN. Through the rational design of mesoporous architectures, strategic immobilization approaches, and enhanced mass transport, substantial improvements in catalytic efficiency and stability are achieved, enabling successful scale-up of reactions from gram to kilogram scales.

### Porous Gels for Continuous Flow Catalysis

Porous gel materials have attracted significant attention due to their tunable pore structures, high specific surface area, and favorable mass transport properties. Particularly, porous gels with hierarchically porous structures enable efficient and stable catalytic conversions in continuous flow systems. In continuous flow catalysis, traditional packed-bed reactors often face challenges such as mass transfer limitations, high pressure drops, and catalyst leaching.

**Fig. 12:**
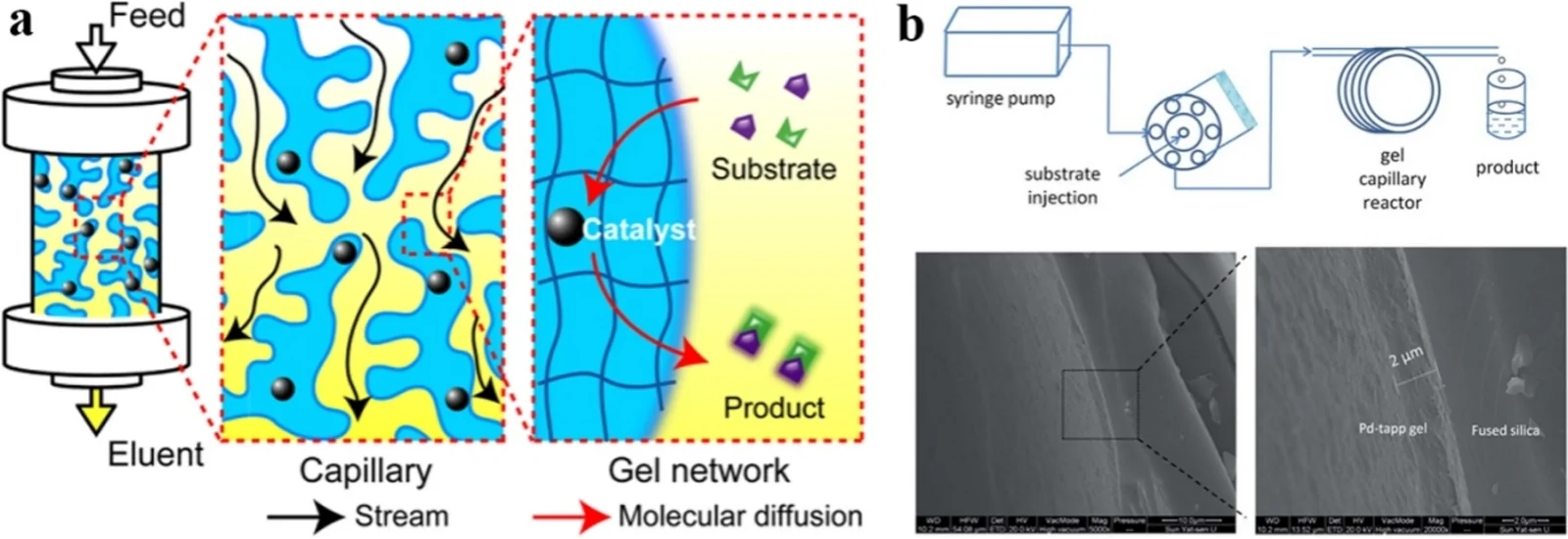
Porous gels for continuous flow catalysis. **a** Conceptual illustration of a flow reactor using monolithic porous gel (MPG) for the catalytic reaction [96]. Copyright 2017, American Chemistry Society. **b** Schematic representation of the setup of the catalytic gel microfluidic reactor using a capillary and SEM images of a cross section of the capillary (ID 0.53 mm) coated with the Pd-tapp-A4 gel with about 2 mm thickness (bars represent 10.0 and 2.0 mm from left to right) [97]. Copyright 2016, Royal Society of Chemistry

To address these issues, Matsumoto et al. [96] developed a monolithic porous gel (MPG) with interconnected capillary pore structures. The column was equipped with 0.45 nm support filters at both the inlet and outlet to retain the catalyst and ensure uniform fluid distribution. This material was synthesized via a one-step copolymerization process, combining macroporous channels with a gel network to provide dual mass transport pathways (Fig. 12a). The internally interconnected capillary pores endowed the material with excellent permeability (permeability coefficient: 10^–14^-10^–13^ m^2^), enabling it to maintain a low pressure drop even under flow conditions. More importantly, the gel network serves as a permeable reaction platform, facilitating rapid diffusion of reactant molecules within the gel phase, thereby enhancing catalytic efficiency. When Pd(0) was loaded into the MPG for the Suzuki coupling reaction, the TON reached 2631 after 30 days of continuous operation, significantly higher than that of catalysts supported on porous glass membranes (TON = 65), silica gel particles (TON = 144), or activated carbon (TON = 26). Furthermore, the confinement effect of the gel network in MPG effectively suppressed metal leaching, demonstrating excellent stability. By adjusting the amount of crosslinker *N,N'-*methylenebisacrylamide (BIS) from 5 to 30 mol%, MPGs with varying network sizes of 2.8–7.0 nm were prepared. As the network size increased, the diffusion coefficients of the reactants phenylboronic acid and 4-bromobenzoic acid, as well as the product 4-phenylbenzoic acid, within the gel phase significantly improved. Correspondingly, the TOF increased from 7.8 h^−1^ for Pd/MPG3 to 27.4 h^−1^ for Pd/MPG1. This indicates that optimizing the gel network structure can enhance molecular mass transport within the gel phase, thereby improving the overall reaction kinetics.

Another representative class of hierarchically porous gels is metalloporphyrin imine aerogels. Zeng et al. [97] synthesized a series of porphyrin imine gels via dynamic imine chemistry, which exhibit a sponge-like three-dimensional network structure composed of interconnected nanoparticles, with high specific surface areas (up to 719 m^2^ g^−1^) and large pore volumes (up to 2.60 cm^3^ g^−1^). These materials not only demonstrate good adsorption performance for gases such as CO_2_ but can also be functionalized with metal centers like Pd(II) to construct catalytic gel microreactors (Fig. 12b). By in situ gelation modification of an amine-functionalized capillary inner wall with Pd-tapp-A4 gel, a catalytic layer approximately 2 μm thick was formed, assembling into a tubular gel capillary reactor. In the Suzuki coupling reaction, this reactor achieved 85% yield within 30 min at 100 °C, outperforming both the homogeneous Pd-tapp catalyst (15%) and batch gel catalytic reactions (34%) under the same conditions. The superior performance is attributed to the efficient mass transport pathways provided by the hierarchical pore structure of the gel and the large specific surface area contact interface offered by microfluidic technology.

### Monoliths for Continuous Flow Catalysis

Monolithic porous materials, as advanced architectures with hierarchically structured pore networks, have demonstrated significant potential in continuous flow catalysis. Their unique three-dimensional interconnected pore systems synergistically combine the mass transport advantages of macropores (> 50 nm) with the high surface area characteristics of mesopores and micropores, effectively addressing limitations inherent to conventional packed-bed reactors such as high pressure drops and mass transfer restrictions. Based on the matrix composition, monoliths can be categorized into silica-based, organic polymer-based, metal oxide-based, and MOF-based types, each exhibiting distinct features in continuous flow catalytic applications.

#### Silica-Based Monoliths

**Fig. 13:**
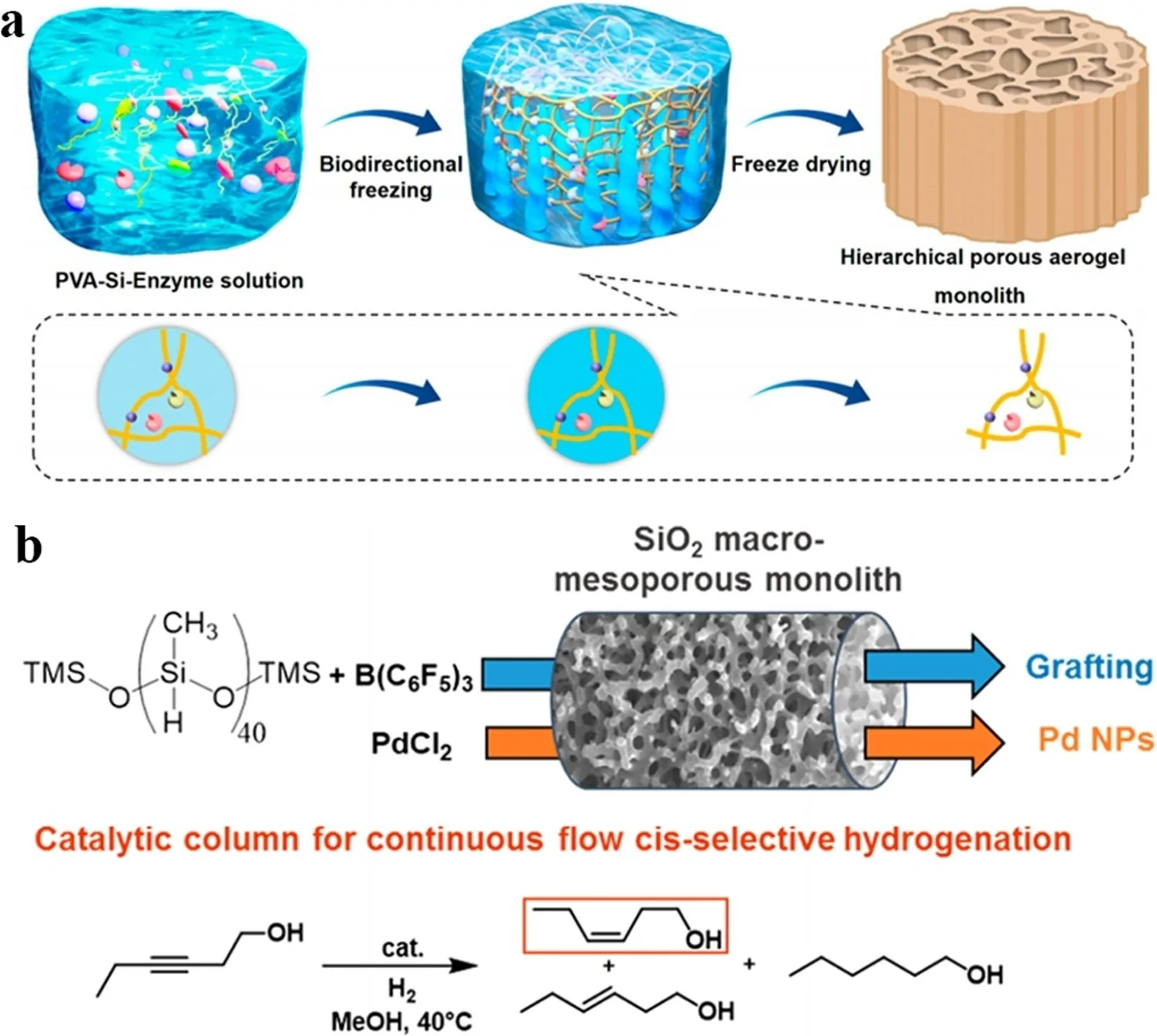
Monoliths for continuous flow catalysis. **a** Fabrication of HPSAM through directional freezing and freeze-drying of PVA-Si-enzyme hydrogels [98]. Copyright 2025, American Chemistry Society. **b** Grafted polymethylhydrosiloxane on hierarchically porous silica monoliths [101]. Copyright 2016, American Chemistry Society

Silica-based monoliths have been extensively investigated as support materials due to their excellent mechanical stability and tunable pore structures. Deng et al. [98] developed a directional freezing–freeze-drying technique to fabricate hierarchically porous silica aerogel monoliths (HPSAM) featuring through macropores of 10–20 µm (Fig. 13a). Structurally, these materials possess a multiscale pore network ranging from micrometer-sized flow-through macropores to nanometer-sized mesopores. This architecture not only facilitates low-resistance transport of reactant fluids but also significantly enhances catalyst loading capacity and the contact efficiency between substrates and active sites. Compared to traditional packed-bed reactors, such monoliths effectively circumvent issues of high pressure drop and diffusion limitations associated with particle packing, while simultaneously overcoming the key bottleneck of low enzyme loading in wall-coated microreactors. The HPSAM continuous flow reactor was prepared via a sol–gel process combined with directional freezing and freeze-drying. The introduction of polyvinyl alcohol (PVA) not only reinforced the mechanical stability of the monolith but also enabled efficient enzyme encapsulation and stable immobilization through the formation of a hydrogen-bonding network with silanol groups. This reactor maintained a low pressure drop even at flow rates up to 20 mL/min, demonstrating excellent fluid permeability.

In the realm of chemical catalysis, Bolton et al. [99] prepared silica monoliths via a sol–gel method and immobilized a 1,10-phenanthroline-Pd complex for Suzuki–Miyaura coupling reactions. In a capillary microreactor with an internal diameter of 250 µm, the reaction between iodobenzene and p-tolylboronic acid achieved a conversion of 68%, maintaining stable activity over 4 days of continuous operation. Turke et al. [100] reported the post-synthetic functionalization of the monolith in the flow phase using (3-aminopropyl)trimethoxysilane (APTMS) successfully introduced amino groups onto the pore surface, creating catalytically active sites. Notably, the choice of solvent during the functionalization process exerted a significant influence on the spatial homogeneity of catalyst distribution. For example, when ethanol was used as the solvent, APTMS achieved uniform dispersion through hydrogen-bonding interactions, leading to consistent grafting along both the axial and radial directions of the monolith. In contrast, the use of toluene resulted in a pronounced gradient distribution, where the catalyst loading reached 1.98 mmol g^−1^ at the front section of the monolith, compared to only 1.08 mmol g^−1^ at its distal end. The resulting functionalized silica monolith demonstrated excellent catalytic efficiency and robust stability in Knoevenagel condensation reactions. At a flow rate of 0.05 mL min^−1^, the system achieved a reaction conversion of 92% under a minimal back pressure of merely 1 bar. Even when the flow rate was elevated to 1.0 mL min^−1^, a conversion of 20% could still be maintained with a back pressure of only 7 bar. Furthermore, by connecting two monolithic reactors in series, the product yield per unit time was enhanced by approximately 15%, underscoring the scalability of this strategy in continuous flow systems. Similarly, Pélisson et al. [101] reported a method involving the grafting of polymethylhydrosiloxane (PMHS) onto hierarchically porous silica monoliths, followed by immobilization of Pd nanoparticles. The resulting Pd@GPMHS40 monolith contained uniformly distributed Pd nanoparticles with a size of 15 nm (Fig. 13b). It achieved 100% conversion in the hydrogenation of styrene, and in the selective hydrogenation of 3-hexyn-1-ol, an alkene selectivity of 88% was attained at 80% conversion, with a space–time yield of 354 g L^−1^ h^−1^. These studies indicated that through hierarchical pore structure design and surface functionalization strategies, silica-based monolithic materials can significantly enhance the mass transfer efficiency and catalyst stability in continuous flow catalytic systems, achieving low pressure drop, high conversion rates, and scalable catalytic processes.

#### Organic Polymer-Based Monoliths

**Fig. 14:**
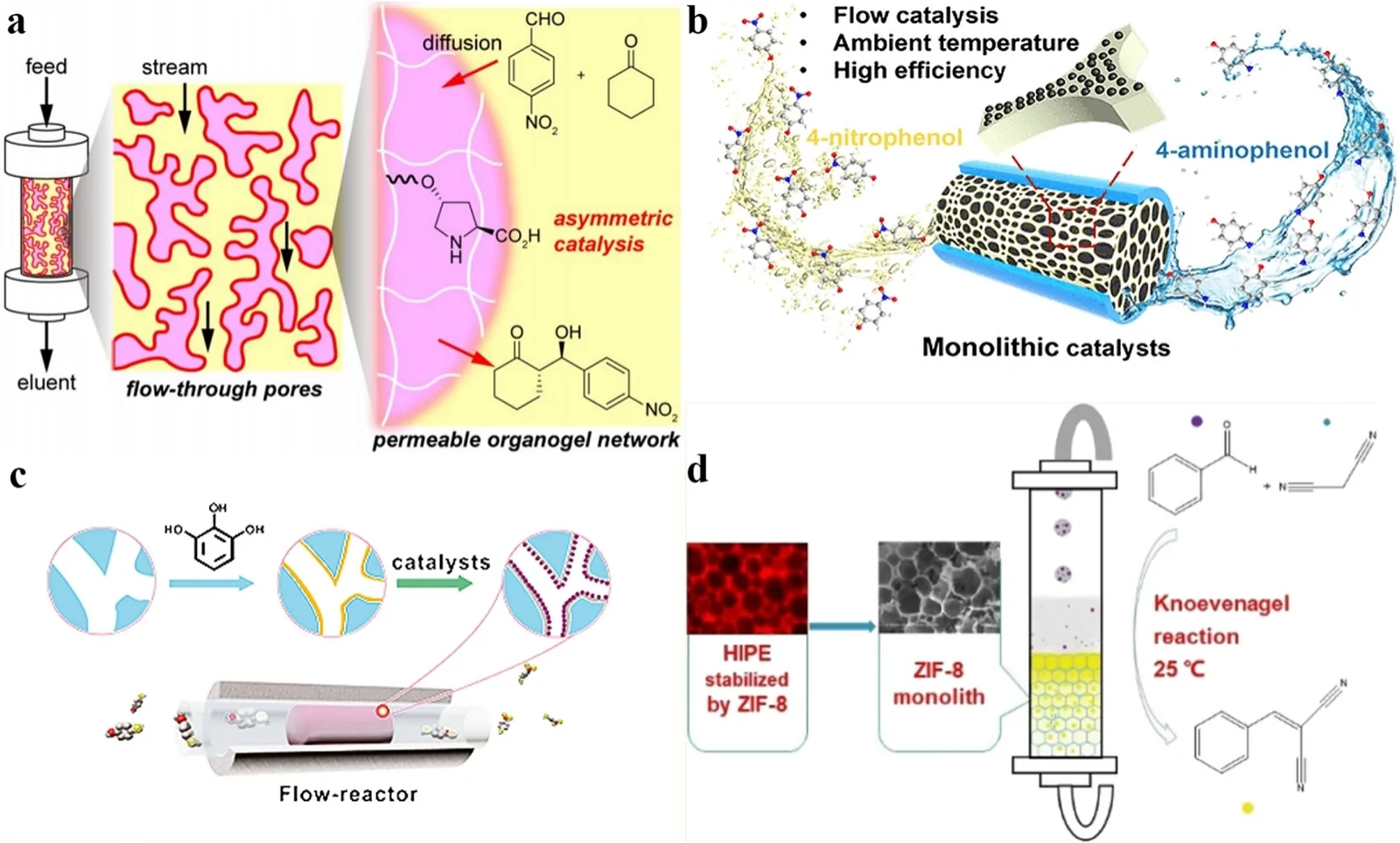
Monoliths for continuous flow catalysis. **a** Schematic of organogel-based porous monolith reactor as continuous flow immobilized catalyst [102]. Copyright 2024, Society of Chemical Engineers, Japan. **b** Monolithic catalysts supported by emulsion-templated porous polydivinylbenzene for continuous reduction of 4-nitrophenol [103]. Copyright 2024, American Chemistry Society. **c** A general, green chemistry approach for immobilization of inorganic catalysts in monolithic porous flow reactors [105]. Copyright 2015, American Chemistry Society. **d** Fabrication of hierarchical macroporous ZIF-8 monoliths using high internal phase pickering emulsion templates [106]. Copyright 2021, American Chemistry Society

Organic polymer-based monoliths are characterized by their abundant surface functional groups and flexible modification strategies. Matsumoto et al. [102] developed an organic gel monolithic reactor by immobilizing L-proline on a poly(butyl methacrylate-co-ethylene glycol dimethacrylate) matrix. Reactant solutions were propelled through the monolith via a syringe pump. The system, equipped with pressure sensors and temperature control units to ensure controlled reaction conditions, was applied to asymmetric aldol addition reactions (Fig. 14a). Under continuous flow conditions, the reaction between p-nitrobenzaldehyde and cyclohexanone proceeded with over 90% conversion and 96% ee. The TON reached 36, significantly higher than the 16 TON observed in batch reactions, attributed to the mitigation of product inhibition under flow conditions. Chen et al. [103] fabricated polydivinylbenzene monoliths via an emulsion templating method. After nitration–amination modification, silver nanoparticles (Ag NPs) were loaded onto the monolith for the catalytic reduction of 4-nitrophenol (Fig. 14b). This monolith exhibited a high apparent rate constant (0.0093 s^−1^) in static catalysis and maintained a 99% conversion for over 2 h in a continuous flow reaction, retaining 90% conversion after 24 h, demonstrating good long-term stability.

#### Metal Oxide-Based Monoliths

Metal oxide-based monoliths have garnered attention due to their excellent thermal stability and unique metal–support interactions. Linares et al. [104] reported a TiO_2_ monolith with a bimodal pore structure. This material exhibits uniform structure, high mechanical strength, and good chemical and thermal stability, overcoming issues such as solvent swelling and poor temperature resistance associated with polymer monoliths. Utilizing such monoliths as supports for immobilizing Pd, Pt, and other metal nanoparticles enables the construction of efficient continuous flow catalytic microreactors. Employing a green in situ synthesis strategy, Pd nanoparticles with an average size of approximately 5 nm were uniformly distributed within the skeleton of the TiO_2_ monolith, achieving a Pd loading of about 0.24 wt%. This catalyst was used under continuous flow conditions for the partial hydrogenation of various unsaturated compounds, including cyclohexene, 1,5-cyclooctadiene, and 3-hexyn-1-ol, demonstrating excellent catalytic activity, selectivity, and long-term stability. Under mild conditions (room temperature, low H_2_ pressure of 2 bar, and short residence times of 40–80 s), reaction conversions reached 60%-90% with selectivities of 63%-95% toward the desired partially hydrogenated products. Notably, the catalyst maintained > 99% of its initial activity after three days of continuous operation, achieving a cumulative TON exceeding 125,000 without detectable Pd leaching, highlighting its exceptional durability. Wang et al. [105] developed a green chemistry approach utilizing pyrogallol (PG) to assist in the immobilization of inorganic catalysts onto HPS monoliths (Fig. 14c). This method successfully enabled the loading of various catalysts such as Au nanoparticles, Al_2_O_3_, and phosphotungstic acid (HPW), applied respectively in nitro compound reduction, Knoevenagel reactions, and esterification. The Au/HPS catalyst achieved 100% conversion in the reduction of 4-nitrophenol, while HPW/HPS reached 92% conversion in ethyl acetate synthesis.

#### MOF-Based Monoliths

MOF-based monoliths combine the high surface area of MOFs with the favorable hydrodynamic properties of monolithic structures. Sun et al. [106] pioneered the use of ZIF-8 nanoparticle-stabilized high internal phase Pickering emulsions as templates to successfully fabricate ZIF-8 monoliths with hierarchical macroporous structures. The present method eliminated the need for polymeric binders, as self-bonded structures were formed through the in situ growth of ZIF-8 particles within the aqueous phase of the emulsion (Fig. 14d). Subsequent freeze-drying yielded ZIF-8 monoliths exhibiting high porosity, ranging from 86.1% to 91.1%. A simple continuous flow catalytic reaction setup was constructed by packing the ZIF-8 monolith into a chromatographic column. This device operated under gravity-driven flow, eliminating the need for external pumps and showcasing its efficient transport performance under low pressure drop conditions. In the Knoevenagel condensation reaction under continuous flow, the reaction between benzaldehyde and malononitrile achieved 100% conversion within 130 s, significantly outperforming the catalytic efficiency of ZIF-8 nanoparticles (3 h) and HMIM (10 min).

### Polymer Composite Materials for Continuous Flow Catalysis

Polymer-based composite materials provide a new approach for continuous flow catalysis. Darder et al. [107] prepared a macroporous polymer monolithic support with ZIF-8 coating using a two-step method guided by nanoparticles. The support was prepared by polymerizing a polymer mixture containing ZnO nanoparticles in a column, and then the surface ZnO was converted into ZIF-8 coating through a dissolution precipitation reaction, increasing the specific surface area from 72 to 273 m^2^ g^−1^. It was used as a capillary microreactor to catalyze Knoevenagel condensation reaction, with a conversion rate of 97% and stable activity in 18 h of continuous reaction. At the same time, the whole body can also be used as an extraction column for rapid pre concentration of trace organic pollutants such as chlorophenols in environmental water, with low back pressure and good flow performance, suitable for low pressure automated processes. Its preparation method has mild conditions and wide adaptability.

## Analysis of Continuous Flow Technology

### Comparison between Continuous Flow Reaction and Batch Reaction

In recent years, the application of such materials in continuous flow reactors has become increasingly widespread. We have summarized key performance metrics, including yield, TON, and stable operation duration, for several representative porous materials under continuous flow conditions (Table 4). Additionally, we have conducted a comparative analysis of the catalytic performance, mass transfer characteristics, and recyclability of porous materials in both continuous flow and batch reactor modes, aiming to provide a theoretical basis for the design of catalytic reactors (Tables 5 and 6).

**Table 4:** Key performance metrics of various porous materials in continuous flow reactions

Catalyst	Reaction type	Yield	TON	Runtime	Refs
ZrOTf-BTC (MOF)	Diels–Alder reaction	93%	1600	17 h	[28]
Pd-BTTA-COF (COF)	Photocatalytic H_2_O_2_ synthesis	968 μmol·h⁻^1^	–	> 100 h	[41]
PP-COP-4 (COP)	Photocatalytic H_2_O_2_ synthesis	2758 μmol·h⁻^1^	–	9 h	[46]
Cage 2 (Cage)	Synthesis of DHQZ	95%	16,000	400 h	[71]
Rh@HZSM-5 (Zeolite)	CO_2_ hydrogenation	68.2%	–	150 h	[81]
Fe_2_O_3_/AlZn-SBA (Mesoporous Silica)	Toluene alkylation	88%	–	3 min	[84]
g-C_3_N_4_ (PCN)	NADH regeneration reaction	82.2%	–	40 min	[92]
MPG (Porous gel)	Suzuki coupling	–	2631	30 days	[96]
Proline-supported monolith (Monolith)	Asymmetric aldol addition	96%	36	3 h	[102]

**Table 5:** Comparison of yields between batch reactors and continuous flow reactors

Reaction type	Catalyst	Yield in Batch reaction (%)	Batch reaction time	Space–time yield (STY) of batch reaction (g g_cat_^−1^ h^−1^)	Yield in Flow reaction (%）	Flow reaction time or flow rate	Space–time yield (STY) of flow reaction (g g_cat_^−1^ h^−1^)	Refs
Halogen exchange reaction	Ni@COF1	77	2 h	0.736	81	–	–	[53]
Decarboxylative fluorination reaction	TpAQ-COF	77	24 h	0.509	82	Efficiency at flux 2000 LMH	17.64	[54]
Reduction of 4-nitrophenol	Ag NPs@SCOF	99	5 min	0.472	> 99	Efficiency at flux 2000 LMH	475.4	[57]
Hydrogenation of nitrobenzene to aniline	Pd/TP-COF	82	1 h	0.0687	94	1 min	4.7	[63]
Enzymatic esterification (hexanol and hexanoic acid)	CALB@COF-MCs-SH	95	2 h	39.87	97	Steady over 72 h	62.1	[64]
1-Phenylethanol (PE) esterification	DMAP@30 nm-SiO_2_	93	150 min	6.49	55	Flow velocity 0.75 mL min^−1^	2.84	[85]
α-Tocopherol (TP) esterification	DMAP@30 nm-SiO_2_	60	150 min	2.56	50	Flow velocity 0.1 mL min^−1^	0.26	[85]

**Table 6:** Characteristics comparison of batch reactors and continuous flow reactors

Characteristic	Continuous flow reactor	Batch reactor	Key implications
Operation mode	Continuous	Batch	Flow reactors operate like an assembly line, with constant feed and product output. Batch reactors complete one cycle at a time
Mixing & heat transfer	Extremely efficient	Less efficient, relies on agitation	The high surface-area-to-volume ratio in flow reactors enables rapid heat exchange and mass transfer, allowing for precise temperature control
Process control	Precise, high reproducibility	Less precise, potential batch-to-batch variation	Stable control of parameters in continuous systems ensures each fluid element undergoes an identical process, leading to consistent product quality
Safety	High (Small reaction volume)	Lower (Large reaction volume)	A core advantage of flow chemistry. The small internal volume minimizes the amount of hazardous material present at any time
Catalyst handling	Typically heterogeneous (fixed bed), difficult to replace	Easier addition and separation	Catalysts are often immobilized within the reactor channels. Batch reactors allow for simple addition and filtration of catalysts
Solid tolerance	Low, prone to clogging	High	A major limitation of microstructured flow reactors. Batch reactors are well-suited for reactions involving slurries or precipitates
Residence time	Short and defined (Seconds to min)	Highly flexible (H to days)	Residence time is fixed by the reactor volume and flow rate. Batch reactors can accommodate reactions requiring very long durations
Capital cost	Higher	Lower	Flow reactor systems require precision engineering, pumps, and control systems, leading to a higher initial investment
Scale-up strategy	Numbering-up, relatively linear	Scale-up, nonlinear challenges	Scaling flow production involves running multiple identical reactor units in parallel, a more predictable process than increasing the size of a single batch vessel

### Comparison and Analysis from the Perspective of Reaction Mechanisms

#### Mixing and Concentration Distribution Mechanisms

In batch reactors, reactant concentrations gradually decrease over time, typically causing the reaction rate to decline as conversion increases. Although the system is fully mixed and spatially uniform in concentration without steady-state gradients, continuous flow reactors establish concentration gradients along the flow direction. Particularly under plug flow reactor (PFR) conditions, the flow approximates a piston-like progression, allowing materials at different conversion levels to be spatially separated. This facilitates better control over intermediates in series reactions and optimization of selectivity. For multiphase catalytic reactions limited by mass transfer, the fixed catalyst bed or wall-coated structures in continuous flow systems provide stable three-phase contact interfaces, reducing fluctuations in mass transfer caused by uneven stirring.

#### Mass and Heat Transfer Mechanisms

Continuous flow reactors, especially microchannel or fixed-bed reactors, possess high specific surface areas and short diffusion paths. Molecular transport is dominated by convective diffusion, resulting in significantly higher mass transfer coefficients compared to mechanically stirred batch tanks. This is particularly critical for gas–liquid–solid three-phase reactions employing porous catalysts, as external diffusion resistance can be substantially reduced, thereby enhancing the apparent reaction rate. Regarding heat transfer, continuous flow systems benefit from a large surface-area-to-volume ratio and forced convection, which effectively suppress the formation and accumulation of local hot spots. This promotes safe operation for highly exothermic reactions and improves selectivity control for temperature-sensitive products.

#### Reaction Kinetics and Pathway Control

Continuous flow systems exhibit a narrow residence time distribution (RTD), closely approaching ideal plug flow behavior, where all fluid elements experience nearly identical reaction histories. Consequently, side reactions accumulate less, and product consistency is higher. In contrast, batch reactors have a broader RTD, which may lead to local overreaction or mixing dead zones, resulting in reduced selectivity. Furthermore, continuous flow allows precise control of reaction time by adjusting flow rates, making it suitable for rapid intermediate capture or optimization of consecutive reactions. Batch reactors, however, are more appropriate for systems requiring extended reaction durations or dynamic monitoring of reaction progress.

### Advantages and Disadvantages of Continuous Flow Technology

#### Key Evaluation of Advantages and Disadvantages of Continuous Flow Reactor

##### Advantages

(I) Exceptional process intensification with enhanced mass and heat transfer. Continuous flow reactors exhibit a significantly high specific surface area, which substantially enlarges the interfacial contact area between reactants and the porous catalysts immobilized on the channel walls or within the packed bed. This configuration markedly reduces the mass transfer distance and promotes the diffusion rate of reactants toward the active sites of the catalyst. Furthermore, continuous flow systems facilitate rapid and uniform heat exchange, a critical feature for managing highly exothermic reactions or processes involving temperature-sensitive compounds. As a result, they contribute to improved reaction selectivity, suppression of undesired side reactions, and prolonged service life of porous catalysts.
(II) Enhanced operational safety. Owing to the minimal reactor holdup, continuous flow systems considerably mitigate the risks associated with runaway reactions. This attribute allows safe operation under severe conditions such as elevated temperatures and pressures. Moreover, such systems enable the secure handling of unstable intermediates or hazardous substances, thereby enhancing overall process safety.
(III) Precise control over reaction parameters and high reproducibility. Through accurate regulation of flow rate, temperature, and pressure, flow reactors allow for exact control of residence time. This facilitates optimization of the reaction pathway and ensures consistent, reproducible, and predictable outcomes, which is essential for both research and industrial applications.
(IV) Facilitation of process integration and automation. The modular nature of continuous flow setups allows straightforward integration with online analytical technologies, enabling real-time reaction monitoring and automated feedback control. The system supports full automation and sustained continuous operation, thereby simplifying long-term catalyst durability tests and enhancing the assessment of process stability.

##### Disadvantages

(I) Challenges in catalyst immobilization and system compatibility. A central challenge in applying porous materials within continuous flow systems lies in the effective immobilization of powdered catalysts. Procedures such as loading, packing, or pre-coating catalysts onto channel surfaces often involve complex steps, which can result in undesirable pressure drops, channel clogging, and potential structural degradation of the catalyst.
(II) High initial investment and operational costs. The initial capital investment required for continuous flow systems, including encompassing pumps, reactors, back-pressure regulators, and control units, generally surpasses that of batch reactors of comparable capacity. In the context of small-scale, multiproduct manufacturing or research applications, the operational flexibility and cost efficiency of batch reactors may present more favorable alternatives.
(III) Limitations in handling solids and risk of blockage. Reactions that generate solid by-products or precipitates pose a significant risk of clogging in microchannels or fixed-bed reactors, potentially leading to operational interruptions. In contrast, batch reactors generally exhibit greater tolerance toward the presence of solid materials.
(IV) Constrained reaction residence time. Residence time in continuous flow systems is governed by reactor volume and flow rate. Achieving extended residence times often necessitates the use of large reactor volumes or very low flow rates, which may be impractical from both technical and economic perspectives. Consequently, for slow reactions, batch reactors may remain the more viable option.

## Catalyst Immobilization Strategies, Hierarchical Design, and Selection of Porous Material Catalysts

### Catalyst Immobilization Strategies

In continuous flow catalysis systems, the selection of immobilization strategies for porous catalysts is of paramount importance, as it is influenced by subtle differences in reaction characteristics and closely related to the unique properties of the catalysts themselves. Currently, the growth, packed bed, and coating method serve as three mainstream immobilization techniques, each demonstrating distinct performance advantages in different application scenarios [108–110]. Tables 7 and 8 systematically summarize the comprehensive impact of these methods on hydrodynamic behavior, catalytic efficiency, and process scalability.

**Table 7:** Comparison of different catalyst shaping strategies in terms of pressure drop, mass transfer efficiency, mechanical/chemical stability, active loading, and scalability

Strategies	Pressure drop	Mass transfer efficiency	Mechanical/chemical stability	Active loading	Capacity scalability
Growth method	Very low	Good (depends on pore structure)	High	High	Moderate (forming process is key)
Packed-bed method	Moderate	High (with small particles)	High	High	High (mature chemical engineering technology)
Coating method	Very low	Moderate (limited by diffusion)	Moderate (risk of coating peeling)	Relatively low	Moderate (challenge of coating uniformity)

**Table 8:** The impact of material shaping strategies on flow behavior and catalytic performance in continuous systems

Strategies	Morphology	Key features	Flow characteristics	Catalytic performance
Growth method	Monolithic structure	The pore structure is designed as an integrated entity, enabling multiscale pores	Low pressure drop, regular and controllable flow channels, conducive to uniform distribution	High accessibility of active sites. Structural robustness affects long-term stability. Suitable for high-throughput, highly exothermic reactions
Packed-bed method	Regular particles	Particle density, strength, and internal pore structure can be controlled	Uniform bed packing with controllable permeability due to regular particle shapes	Internal diffusion resistance is a key limiting factor. Porosity and pore size distribution determine the mass transfer efficiency of reactants to internal active sites
Coating method	Thin film	Coated onto pre-formed macroscopic supports	Coating thickness and morphology determine the surface flow boundary layer and local mass transfer	High utilization of active components, excellent mass and heat transfer efficiency. The adhesion between the coating and the support is crucial

The growth method involves synthesizing catalysts directly within microfluidic channels, providing an effective approach for achieving efficient, precise, and controllable immobilization of heterogeneous catalysts. The primary advantage of this method lies in its ability to fully utilize the unique microenvironmental characteristics of microchannels, such as high specific surface area, rapid mass transfer rates, and precise control over reaction conditions, thereby promoting efficient and uniform synthesis and growth of catalyst particles. This technique is particularly suitable for reaction systems requiring long-term operational stability, high product selectivity, and strict energy consumption control [111–113]. When selecting this method, key considerations include the specific surface area and mass transfer efficiency of the microchannels, the capability for precise control of reaction parameters (e.g., temperature, pressure, flow rate), the compatibility between the catalyst material and microchannel growth.

The packed-bed method achieves efficient utilization of catalysts by packing catalyst particles with non-uniform morphologies into the channels of microfluidic reactors. This approach offers core advantages such as low cost, ease of operation, and broad applicability, making it particularly suitable for reaction systems catalyzed by low-cost particulate catalysts and for gas–liquid–solid three-phase reactions [114–117]. When adopting the packed-bed method, it is essential to systematically evaluate the following three key aspects: (Ⅰ) microchannel reactor characteristics, involving structural compatibility with the particle system and volumetric capacity; (Ⅱ) particle morphological parameters, including catalyst size, geometric configuration, and spatial distribution patterns; (Ⅲ) implementation processes, ensuring uniform dispersion of the catalyst during packing and operational adaptability under various process parameters. Through comprehensive assessment of these aspects, optimal catalytic performance can be achieved while maintaining structural integrity in dynamic reaction environments.

The coating method primarily employs techniques such as catalyst mixed slurry coating and chemical vapor deposition to form uniform and stable catalyst layers on the inner walls of microfluidic reactors. This approach encompasses steps including the selection of precursor materials, mixture/deposition, and optimization of the catalyst layer, aiming to provide excellent specific surface area and rapid molecular diffusion capabilities [118, 119]. It is particularly suitable for applications requiring significant enhancement of catalyst activity, especially in high-value catalyst systems. Therefore, selecting appropriate precursor materials is crucial to ensuring the quality and performance of the catalyst layer, while considerations such as the chemical properties of the microchannels, compatibility with the mixture/deposition process, and the potential improvement in catalyst layer performance must also be integrated.

In summary, the selection of immobilization techniques for porous powders should follow a systematic decision-making process. Firstly, conducting a comprehensive analysis of key factors such as reaction type and conditions. Secondly, performing an integrated evaluation of the physicochemical properties, activity, and stability of the heterogeneous catalyst. Furthermore, considering the influence of actual operating conditions on the immobilization strategy. On this basis, cost-effectiveness analysis should be carried out to identify the most economically viable immobilization solution. Ultimately, experimental validation of different immobilization strategies is required to determine the optimal immobilization approach.

### Hierarchical Design

The design of hierarchical pore architectures (macro–meso–microporous) represents a key strategy for addressing the core trade-off between mass transfer and pressure drop in continuous flow processes. Compared to purely microporous materials, which possess abundant active sites but suffer from severe diffusion limitations, such multiscale pore systems enhance overall performance directly and significantly through synergistic effects. The underlying mechanism lies in the distinct roles played by each pore level: Macropores drastically reduce pressure drop and ensure uniform fluid distribution across the reactor cross section; mesopores facilitate rapid diffusion of reactants toward active-site regions; while micropores ultimately provide high specific surface area and a dense population of catalytic or adsorption sites. This architecture effectively decouples mass transport from surface reaction processes (Table 9).

**Table 9:** Comparison between hierarchical porous materials and purely microporous materials

Performance metric	Purely microporous materials	Hierarchical porous materials (Macro–Meso–Microporous)	Enhancement mechanism
Mass Transfer Efficiency	Low, controlled by internal diffusion	High, convection-enhanced mass transfer	Mesopores/macropores provide rapid diffusion pathways
Pressure Drop	High, especially at high-flow rates	Significantly reduced	Macropores provide low-resistance flow paths
Active-Site Utilization	Low (often < 30%)	High (can be > 80%)	Shortened diffusion paths render internal sites accessible
Deactivation	Poor, prone to pore-mouth blocking	Strong, facilitates by-product/coke removal	Macropores allow passage and expulsion of particles/large molecules
Kinetic Response	Slow breakthrough	Fast, rapid response	Enhanced apparent diffusion coefficient
Fluid Compatibility	Clean, small-molecule fluids	Viscous fluids, or fluids containing particles/large molecules	Macropores accommodate complex fluid dynamics
System Design Flexibility	Requires large particles or thin layers to avoid pressure drop	Can be designed as monolithic reactors/electrodes for compact systems	Structure combines high surface area with low flow resistance

In the field of continuous flow adsorption separation, the performance contrast is particularly pronounced. Under fast-flow conditions, purely microporous adsorbents require extended time for adsorbates to diffuse into the deep micropores, leading to only the inlet portion of the bed being effectively utilized. This results in sharp breakthrough curves and low dynamic adsorption capacities. Meanwhile, increasing bed height to achieve sufficient contact time causes a dramatic rise in pressure drop and energy consumption. Hierarchically porous adsorbents fundamentally alter this scenario: Macropores allow fluid to pass rapidly through the entire bed with minimal resistance, while mesopores ensure efficient mass transfer between the fluid and the microporous adsorption sites. Consequently, at the same flow rate, hierarchical adsorbents exhibit more gradual breakthrough curves and higher dynamic adsorption capacities, implying greater bed utilization. Molavi et al. [120] utilized 3D printing technology to fabricate an alumina scaffold featuring periodically interconnected macropores of 500 μm. This scaffold served as a host for the in situ growth of microporous ZIF-8, thereby constructing a structured hierarchically porous MOF catalytic reactor. In the continuous flow Knoevenagel condensation reaction of benzaldehyde and ethyl cyanoacetate, this structured reactor demonstrated remarkable performance. Compared to a fixed bed directly packed with ZIF-8 powder, its reaction conversion rate increased by nearly fourfold at the same space velocity, while the pressure drop was less than 1/20 of that in the powder bed. More importantly, no activity decay was observed after 140 h of reaction. In contrast, the powder-bed reactor suffered from a decline in conversion of over 30% after only 60 h due to pore blockage and accumulation of intermediate products caused by internal diffusion limitations. This clearly demonstrates that the introduced macroporous channels not only reduce the pressure drop but also significantly enhance reaction efficiency and catalyst stability by promoting overall mass transfer and facilitating rapid exchange of reactants and products. Chen et al. [121] synthesized hierarchically structured ZSM-5 zeolites with interconnected macropores and micropores via nanocrystal assembly combined with a soft-hard dual-template method. When employed for the continuous flow adsorption of toluene, these hierarchical zeolites exhibited a dynamic adsorption capacity nearly double that of conventional micron-sized ZSM-5 crystals. Notably, the pressure drop across the hierarchical zeolite bed was reduced by 70%, and the breakthrough curve was more gradual, indicating a broader adsorption front and higher bed utilization. The presence of mesoporous channels accelerated the diffusion of toluene molecules to the microporous active sites, thereby mitigating the internal diffusion constraints commonly encountered in purely microporous materials under high gas flow rates.

In summary, the performance improvement offered by hierarchical pore structures in continuous flow is fundamental compared to purely microporous materials. It is not merely an increase in pore volume, but rather a systemic optimization achieved by constructing an interconnected network spanning from the macroscopic to the nanoscale. This approach integrates and optimizes the three critical steps of fluid delivery, diffusion, and surface reaction. It directly addresses the inherent bottlenecks of purely microporous materials, such as diffusion limitations, high pressure drop, susceptibility to clogging, and low site utilization efficiency. As a result, key performance indicators including reaction rates, separation kinetics, energy efficiency, and operational lifespan are simultaneously enhanced. Therefore, hierarchical structural design constitutes the core material foundation for realizing highly efficient, compact, and stable continuous flow processes.

### Selection Decision Matrix

The core of the decision matrix for porous material selection lies in dynamically interrelating material characteristics (e.g., pore size, stability, and functionalization potential) with flow system engineering requirements (such as mass transfer efficiency, pressure drop, and reactor configuration), rather than isolating any single parameter for individual evaluation (Table 10). First, pore size and pore architecture serve as the primary screening criteria. For photocatalytic reactions or transformations involving large molecules, materials with well-defined and sufficiently large pores (e.g., many COFs or mesoporous MOFs) are necessary to ensure adequate contact between reactants and active sites; whereas for gas-phase reactions involving small molecules, microporous materials (e.g., rigid MOFs or zeolites) may be more conducive to selective diffusion. Second, chemical and mechanical stability is critical for the feasibility of continuous flow processes. In environments involving solvents, elevated temperatures, or reactive chemical species (such as radicals, acidic/basic media), priority should be given to highly stable materials (e.g., some Zr-MOFs, COFs with strong covalent linkages, or surface-passivated cage compounds) to avoid material degradation that could lead to system blockage or performance decay. Third, the ease of functionalization determines whether a material can be precisely tailored for specific reactions, for scenarios requiring the introduction of catalytic sites or modulation of hydrophilicity/hydrophobicity, materials amenable to post-synthetic modification or possessing inherent functional groups (e.g., amine-containing COFs or MOFs with tunable metal nodes) offer distinct advantages, though the impact of modification on structural integrity must be carefully weighed. Finally, compatibility with the geometry of the flow reactor, though often overlooked, is essential. Materials must be integrable in appropriate forms (e.g., monolithic columns, coatings, or shaped pellets) into microchannels, fixed beds, or membrane reactors, which demands good processability (e.g., COPs that can be fabricated into thin films or sintered MOFs with high mechanical strength) while maintaining high permeability and low flow resistance.

**Table 10:** Decision matrix for selecting porous materials in continuous flow systems

Types	Pore size	Stability	Functionalization and activity	Compatibility with flow reactor geometry
Photocatalytic applications	Sufficiently large pores are required to accommodate substrate molecules and facilitate product diffusion; ordered channels benefit light propagation and active-site exposure	Must withstand reaction solvents, potential photogenerated reactive oxygen species, and reaction conditions	Facile integration of photosensitizing groups or semiconductor units; band structure must be matched	Microreactor, fixed bed
Gaseous phase/small-molecule catalysis	Micropores (< 2 nm) are usually sufficient; pore surface chemistry and size selectivity are critical	Must remain stable under high reaction temperatures and be inert to reactants/products	Ease of introducing high density, highly selective catalytic active sites	Fixed bed, monolithic column
Multiphase flow systems	Relatively large mesopores (> 2 nm) are needed to promote rapid transport of liquid reactants and products, preventing clogging	Must tolerate flowing solvents, possible acidic/alkaline environments, and long-term continuous operation	Facile surface hydrophilic/hydrophobic modification or anchoring of specific catalysts to optimize interfacial reactions	Slurry reactor, membrane reactor

In summary, the selection process should be treated as a multi-objective optimization. For instance, in photocatalytic flow systems, materials with broad-spectrum light absorption and ease of fabrication into transparent thin films may be prioritized; for multiphase catalytic flow reactions, a balance must be struck among active-site density, hydrothermal stability, and pellet-forming capability. This decision matrix is not a static checklist but an iterative framework that requires combining simulation predictions with experimental validation. The ultimate goal is to achieve synergy among material properties, reaction kinetics, and flow engineering parameters, thereby enhancing the efficiency and sustainability of continuous flow processes.

## Development Trends of Continuous Flow Systems for Porous Catalysts in Industrial-Scale Applications

### New Strategies for Preparing Porous Catalysts

In the domain of catalyst material design, surface coating and composite structure construction have demonstrated significant effectiveness. For instance, the in situ growth of COF-TpBpy on silica gel followed by coordination with copper(I) to form SiO_2_@CuI-TpBpy substantially enhances metal loading stability and reduces internal diffusion resistance. The resulting fibrous porous structure also alleviates the influence of particle size on pressure drop and reaction yield [56]. Similarly, the immobilization of MOFs, such as MIL-100(Sc) and ZrOTf-BTC, onto PBSAC or SiO_2_ supports, which are characterized by high mechanical strength and uniform particle sizes, addresses common issues including clogging and excessive pressure drop associated with powdered catalysts, thereby facilitating long-term operation in continuous flow systems [28, 34]. In the context of support structure optimization, the design of porous monoliths, for example M-PS-TPP, or HPSAM exhibiting high porosity, ranging from 81% to 98%, and large pore sizes of 1.0 and 3.6 μm, significantly improves mass transfer efficiency while minimizing flow resistance, ultimately yielding a residence time distribution that closely approximates ideal plug flow conditions [61, 122].

### Large-Scale Synthesis of MOFs/COFs

Traditionally, the large-scale synthesis of MOFs and COFs faces significant challenges. Their classic hydrothermal or solvothermal methods are typically batch operations, which suffer from inefficient mass and heat transfer, product quality fluctuations upon scale-up, harsh reaction conditions, and long processing times. More critically, batch synthesis offers limited control over crystal size and morphology, which directly determine mass transport and active-site accessibility in catalytic applications. These bottlenecks have driven researchers to explore continuous flow synthesis as a highly promising solution.

Continuous flow synthesis provides a systematic solution to these challenges by enabling precise mixing and condition control within meticulously designed microchannels or reaction setups, primarily including single-phase laminar flow synthesis and multiphase segmented flow synthesis [123–125]. It involves pumping metal salt and organic ligand solutions into micrometer-scale channels, where the extremely high surface-area-to-volume ratio facilitates instantaneous and homogeneous mixing, thereby triggering rapid and uniform nucleation. The core advantage of this method lies in its exceptional controllability: By precisely adjusting flow rates, temperature, and concentration, it allows for the reproducible preparation of small crystals, typically on the nanoscale with uniform size. This not only substantially shortens reaction times but also directly yields ideal catalytic materials possessing high specific surface area and abundant exposed active sites. Rasmussen et al. [126] employed a counter-current mixer (CCM) to optimize the heat and mass transfer between supercritical carbon dioxide (scCO_2_) and MOF precursors. In the continuous flow setup, high-temperature scCO_2_ was delivered through the inner tube of a coaxial structure, while the MOF precursor mixture at ambient temperature flowed through the outer tube, with the two streams moving in counter-current directions. When scCO₂ bubbles exited the inner tube into the precursor liquid in the outer tube, buoyancy caused them to rise, generating intense counter-current shear with the precursor and achieving rapid mixing. This allowed the reaction to complete within an extremely short time (< 3 s). Taking the zirconium-based MOF UiO-66 as an example, this system achieved a production rate of 104 g h^−1^. Traxler et al. [127] designed a continuous flow process consisting of two feed streams: One dissolving aldehyde monomers and the other amine monomers, with benzoic acid added as a catalyst and aniline as a modulator. The two solutions rapidly converged in a Y-mixer before entering a heated reaction tube with a 1/16-inch inner diameter and a volume of approximately 0.5 mL, maintained at a constant temperature of 90 °C. By adjusting the feed pump flow rates, the reaction residence time could be flexibly controlled between 5 s and 6 min, a range far shorter than the h or even days required by conventional solvothermal synthesis. This approach successfully produced micrometer-sized single-crystal COFs, including the hexagonal CF-TAPB-DMPDA and the rhombic CF-TAPPy-PDA, within remarkably brief reaction times. Similarly, Zhao [14] and Khalil et al. [128] reported continuous synthesis methods for imine-linked COFs using coiled flow reactors, respectively, achieving a dual breakthrough in scalable production and processing techniques, with a space–time yield reaching 61,111 kg m^−3^ day^−1^.

The maturation of continuous flow synthesis techniques for MOF/COF materials has laid a solid foundation for their efficient and controlled synthesis. The advancement of such synthetic methodologies enables precise modulation of the material’s structure, morphology, and pore properties, thereby supplying porous materials with reliable performance and batch-to-batch consistency for subsequent applications. This progress naturally leads to a shift in research focus toward the effective integration of these structurally well-defined functional materials into continuous flow catalytic reaction systems. By directly employing MOF/COFs as solid catalysts or catalyst supports packed in continuous flow reactors, it is possible to fully leverage their advantages, such as high specific surface area, tunable active sites, and shape-selective properties. Moreover, such integration aligns with the inherent strengths of continuous flow processes, including enhanced mass and heat transfer, improved process safety and controllability, and ease of scaling and integration. Together, these features pave a new avenue for developing next-generation catalytic processes that are highly efficient, stable, and capable of continuous operation.

### Activation of Porous Catalysts in Industrial Scale-Up

In continuous flow systems, the deactivation of porous catalysts, such as coking, active-site poisoning, pore blockage, or structural changes, severely limits their long-term stable operation. Therefore, in situ regeneration and continuous regeneration technologies that do not require interrupting production or disassembling the reactor have become crucial research directions for achieving efficient and economically viable industrial processes. However, the industrial scale-up of such systems still faces multiple technical challenges. On one hand, the catalyst materials themselves have limitations, including uncontrolled structure, insufficient chemical functionality, limited metal loading capacity, and metal ion leaching, which affect stability and product purity. On the other hand, in reactor operation, powdered materials can easily lead to excessive packed-bed resistance, high pressure drops, and clogging, coupled with issues such as low mass transfer efficiency, deposition of insoluble salts, and inhibition of side reactions, all of which hinder the stable and safe scale-up of continuous flow processes.

To overcome these challenges, various strategies have been developed. In practical operation, for materials with high thermal stability such as zeolites and mesoporous silica, thermal regeneration is the most commonly used in situ method. Daligaux et al. [129] found that in the methanol-to-olefins (MTO) fixed-bed process, a dual-reactor switching system is widely employed, where one reactor performs the catalytic reaction while the other introduces air to burn off coke deposits, enabling pseudo-continuous production. At the laboratory scale in microreactors, periodic programmed switching between reaction feed and oxygen-containing regeneration gas can also achieve pulsed regeneration of zeolite catalysts. For catalysts supported on mesoporous silica in liquid-phase reactions, in situ solvent flushing can be adopted, where the flow is switched to a solvent capable of dissolving deposits (such as ethyl acetate or scCO_2_) without interrupting the process, thereby cleaning the catalyst surface and pores. For materials with relatively lower stability, such as MOFs and COFs, regeneration conditions must be milder. A common strategy is thermal desorption under an inert atmosphere (e.g., using hot nitrogen purging to remove volatile adsorbates) [130, 131]. Furthermore, in electrocatalytic continuous flow systems, applying periodic electrochemical potentials to supported COF catalysts can oxidatively remove toxic intermediates from the surface, restoring their active sites [132–134].

## Conclusion and Perspective

The utilization of porous materials in continuous flow catalytic reactions is underpinned by a robust theoretical foundation and exhibits diversified developmental trajectories, encompassing a variety of structural types including MOFs, COFs, cages, porous silicates, monolithic structures, and PCNs. These materials offer an ideal platform for the efficient loading and precise regulation of catalytic active sites, owing to their high specific surface area, well-ordered pore channels, and chemically tunable surfaces. These characteristics, combined with continuous flow technology, effectively overcome the limitations inherent in traditional catalysis, such as catalyst deactivation, metal leaching, and increased mass transfer resistance, thereby comprehensively enhancing catalytic activity, selectivity, and operational stability.

For MOFs and COFs, advanced structural design strategies coupled with continuous flow synthesis techniques facilitate the precise construction and spatial distribution of active sites through tailored regulation of metal nodes, organic ligands, monomers, and defect engineering. This approach not only enhances utilization of active sites and improved pathway selectivity, but also, in conjunction with optimized pore dimensions and structural homogeneity, contributes to significantly enhanced mass transfer efficiency. Furthermore, porous polymer monoliths and their composite counterparts exhibit superior performance in diminishing mass transfer limitations and promoting the immobilization efficiency of enzymatic or metallic catalytic species, achieved through hierarchically organized porosity and controlled chemical grafting. These characteristics collectively establish such materials as highly efficient and robust supporting platforms for multistep continuous flow catalytic systems.

In terms of catalytic applications, porous material-based catalysts cover cross-coupling, hydrogenation, redox reactions, small-molecule conversion, and photocatalysis fields, all exhibiting excellent catalytic activity, selectivity, and long-term operational stability. Particularly in photocatalysis and photothermal catalysis, optimization of photogenerated carrier separation and reaction pathways has been achieved through functionalized defect engineering, multi-energy field coupling, and donor–acceptor structure regulation, enabling sustainable and efficient catalysis. In biocatalysis, porous supports facilitate the synergistic immobilization of enzymes and cells and the regulation of microenvironments, significantly improving biotransformation efficiency and reaction stability, and driving the development of multistep cascade continuous flow synthesis.

A systematic investigation into the pore architecture, electronic structure, and processing parameters of porous catalysts elucidates their profound impact on reaction–diffusion kinetics, the electronic configuration of active sites, and catalytic reaction pathways. Well-tailored pore size distributions and interconnectivity serve to optimize reactant transport and enhance catalytic site accessibility. Simultaneously, strategic modulation of active-site electronic structure, accomplished through functional group incorporation, defect engineering, and support ligand environment modification, facilitates precise control over reaction selectivity. Additionally, key process variables including temperature and flow rate significantly influence both catalytic efficiency and long-term stability.

In summary, porous materials, with their tunable structural and functional advantages, demonstrate broad application potential and scientific value in the field of continuous flow catalysis. Looking ahead, the following specific research directions and technical challenges warrant focused attention: (I) In terms of material design, efforts should be directed toward developing novel porous materials with excellent hydrothermal stability and mechanical strength, overcoming the long-term stability challenges of MOFs/COFs under continuous flow conditions. Achieving atomic-scale precision in regulating the spatial and electronic structures of active sites on porous supports, alongside advancing corresponding in situ characterization techniques, is crucial. Furthermore, the design of intelligent catalytic materials with stimuli-responsive, adaptive, or self-healing functionalities represents a promising frontier. (II) At the mechanistic level, it is essential to systematically elucidate the dynamic coupling relationships among reactant diffusion, interfacial transport, and catalytic conversion within micro-/mesoporous environments through multiscale simulations combined with in situ experiments. The use of spatiotemporally resolved in situ spectroscopy and microscopy techniques can reveal the dynamic evolution of active sites under reaction conditions. Additionally, in-depth exploration of the synergistic catalytic mechanisms and energy transfer dynamics under coupled physical fields such as light, electricity, and heat, remains a key research priority. (III) At the system and engineering level, the development of efficient, scalable modular continuous flow reactors is necessary to achieve precise control over catalyst packing, temperature regulation, and fluid distribution. Integrating reaction units with separation modules such as membrane separation or adsorption can enable the construction of highly efficient, integrated catalytic systems. Moreover, leveraging machine learning and automation technologies to facilitate intelligent decision-making in catalyst design, process optimization, and system control represents a critical advancement. (IV) At the sustainability level, there is an urgent need to develop low-energy, low-waste green synthesis pathways and efficient catalyst regeneration strategies. Exploring porous catalytic materials derived from biomass or biodegradable polymers can significantly reduce environmental footprints. Furthermore, designing integrated systems for CO_2_ capture and catalytic conversion will drive the practical application of continuous flow catalysis in carbon neutrality initiatives. By focusing on these outlined pathways and challenges, the field can propel continuous flow catalysis toward greater efficiency, intelligence, robustness, and sustainability, ultimately accelerating the transition from laboratory innovation to large-scale industrial implementation.
